# Development of a carbon fiber-based microextraction sample preparation patch for the detection of 21 organochlorine pesticides from water

**DOI:** 10.1038/s41598-026-36604-0

**Published:** 2026-01-28

**Authors:** Harshika Poojary, Chiranjit Ghosh

**Affiliations:** Manipal Institute of Technology, Manipal Academy of Higher Education, Manipal, Karnataka 576104 India

**Keywords:** Sample preparation, Pesticides, Pollutants, Solid-phase microextraction, Green sample preparation, Chemical water pollutant, Organochlorine pesticides, Chemistry, Environmental sciences

## Abstract

The widespread use of organochlorine pesticides (OCPs) in agricultural practices has led to its accumulation in water bodies, causing significant environmental and health risks due to its persistence and toxicity. To enable effective monitoring, carbon fiber-based thin-film solid-phase microextraction (TF-SPME) patch were developed using a uniform divinylbenzene (DVB) coating applied exploiting an automatic film applicator. These patch were tailored for the extraction and quantification of 21 OCPs from water matrices. Extraction parameters such as extraction time, temperature variation study, desorption time and solvent profile were optimized using water samples spiked with a standard mixture of the 21 OCPs across a concentration range of 100–900 ng/mL. The DVB-coated carbon fiber TF-SPME patch exhibited excellent extraction efficiency, achieving limits of detection ~ 0.3–1.5 ng/mL when analyzed with a triple quadrupole gas chromatography-mass spectrometer (GC–MS/MS). Calibration curves with regression equations were established for each pesticide to support rapid and reliable quantification. The method aligns with green analytical chemistry principles due to its minimal solvent consumption, low waste production, and energy-efficient operation. Greenness assessment tools such as AGREE, GAPI, and BAGI confirmed the method’s environmental compatibility and user safety. This DVB-coated carbon fiber TF-SPME platform presents a sensitive, robust, and eco-friendly approach for the routine monitoring of organochlorine pesticides in water resources, crucial for protecting ecological balance and public health.

**Supplementary Information:**

The online version contains supplementary material available at 10.1038/s41598-026-36604-0.

## Introduction

Organochlorine pesticides (OCPs) in water sources are a real concern due to its toxicity persistence, and bioaccumulation in the food chain^1^. These pesticides include (dichlorodiphenyltrichloroethane) DDT, aldrin, heptachlor, and endosulfan, which have been widely used in farming and controlling pests^2^. Even though many countries have banned or restricted the use of toxic pesticides, the residues were still reported in groundwater^3^. Long-term human exposure to these OCPs has been correlated with endocrine disruption, reproductive toxicity, immunotoxicity, neurodevelopmental and neurological effects, as well as elevated risks of cancers and metabolic disorders^1,4,5^. OCPs can penetrate the placental barrier and accumulate in breast milk, thereby heightening the vulnerability of infants and developing fetuses^6^. Monitoring these contaminants at trace levels is essential for environmental safety and public health^7–11^.

Traditional methods, including liquid–liquid extraction (LLE) and solid-phase extraction (SPE) are widely used to extract organochlorine pesticides from water^12^. However, these techniques require prolonged time and necessitate the use of large quantities of toxic solvents that harm the environment, and involve multistep sample preparation processes. Consequently, there has been growing demand for environmentally friendly, miniaturized, and solvent-free extraction techniques that align with the principles of green analytical chemistry (GAC)^13^.

Solid-phase microextraction (SPME) has emerged as a transformative sample preparation technique over the last twenty years^14^. It combines sampling, extraction, concentration, and sample introduction in a single step^15,16^. The primary advantage of this technique is that it requires a minimal amount of solvents^17^. Among its various types, thin-film SPME (TF-SPME) stands out because it offers a larger surface area for capturing analytes, resulting in improved extraction performance and faster equilibrium times^18–24^. Lots of recent research is focused on developing new substrates and coating materials for TF-SPME, especially for environmental testing and cleanup^25–35^.

Researchers developed a special coating made from a β-cyclodextrin-based hyper-crosslinked polymer (β-CD-HCP), and the coating recipe was used to fabricate the glass fiber-based microextraction patch. This coated patch extracted six types of polyaromatic hydrocarbons (PAHs) from water samples when used with in-tube SPME. The method was found to be sensitive, with detection limits of 0.004–0.008 µg/L, as determined by HPLC–DAD analysis. The method also demonstrated good recovery rates, ranging from approximately 80% to 100%, and provided strong enrichment factors. Although the developed method showed good extraction performance, glass fiber was not durable and it tended to break easily. Therefore, this approach was not suitable for reuse after 2–3 cycles^36^. In addition, the researchers developed a paper-based thin-film solid-phase microextraction (TF-SPME) patch for the targeted analysis of 4-chlorophenol (4-CRP), a harmful water pollutant. Researchers fabricated this patch by coating regular cellulose paper with a composite coating comprising DVB/PDMS/MWCNT (divinylbenzene/polydimethylsiloxane/multi-walled carbon nanotubes). When tested by GC–MS, it proved quite effective, with a detection limit of approximately 10 ng/mL and the ability to measure concentrations ranging from 100 to 10,000 ng/mL. The major benefits of this study include the low-cost method along with the requirement of a small quantity of solvent. However, a paper-based patch is not thermally or mechanically stable, which could limit its use for some applications ^19^. To address this issue, a carbon fiber substrate offers a suitable alternative for analytical sample preparation purposes due to its good mechanical stability and cost-effectiveness of the materials used. Therefore, carbon-based material, such as carbon fiber, is a potential platform for fabrication of TF-SPME substrates. Additionally, divinylbenzene (DVB) is a polymer used as a sorbent due to its hydrophobic nature, which naturally attracts nonpolar and semi-volatile organic compounds. Its porous structure and ability to withstand high temperatures make it a perfect tool, especially for detecting pollutants like organochlorine pesticides (OCP)^37–40^.

Over the past few years, the principles of Green Analytical Chemistry (GAC) have been a significant concern in reducing environmental impacts. GAC emphasizes in reducing the use of toxic chemicals, preventing the generation of waste materials, and enhancing the efficiency of analytical processes. Thus, it encourages the development of sustainable alternatives to conventional methods. For the purpose of evaluating the environmental friendliness of such processes, several assessment tools were reported, such as AGREE (Analytical GREEnness Metric Approach and Software), BAGI (Blue Applicability Grade Index), and Complex GAPI (Green Analytical Procedure Index). These assessment methods provide a systematic and quantitative means of evaluating the environmental sustainability of analytical methods, ensuring its compliance with the green chemistry principles^41^.

In this study, we developed DVB-coated carbon fiber microextraction patches to extract 21 different organochlorine pesticides from water samples. The coating combines a carbon fiber sheet with the selective adsorption properties of DVB, creating a sustainable, and sensitive tool ideal for monitoring water quality. We optimized and verified the extraction process using triple quadrupole gas chromatography-mass spectrometry (GC–MS/MS). The environmental friendliness score of this sample preparation technique was calculated through the various assessment parameters, including AGREEprep, BAGI, and GAPI . This approach demonstrated good sensitivity for monitoring OCPs in water samples.

## Materials and methods

### Materials

The organochlorine pesticide stock solution was prepared using the EPA 8081 Pesticide Standard Mix (Supelco, USA). The carbon fiber substrate (200 GSM, plain weave) was obtained from BhorForce PC200 68151200 (Bhor Chemicals & Plastics Pvt. Ltd., Worli, Mumbai, India). Acetonitrile extra pure, 98% and hexane (HPLC grade) were procured from Supelco, USA. Methanol (99% purity) was procured from Finar Ltd. (Ahmedabad, India), and isopropanol (IPA, 99% purity) was obtained from Loba Chemie Pvt. Ltd. (Mumbai, India). Sylgard 186 silicone elastomer kit was supplied by Dow. Divinylbenzene (DVB) monomer was purchased from Sigma-Aldrich (USA), and azobisisobutyronitrile (AIBN, 98%) was procured from LOBA Chemicals (Mumbai, India). The commercial thin-film SPME patch used in our study was obtained from Markes International USA (PDMS/DVB TF-SPME, product code Z-8367, batch no. 640689). All glassware, including 40 mL glass vials with septa and screw caps, was purchased from Borosil. (Mumbai, India).

### Instruments

An Agilent 8990 GC and a 7000E triple-quadrupole MS/MS (Agilent Technologies, U.S.A.) were used for separation and quantitation, respectively. A PAL3 autosampler equipped with a 10 µL syringe (ID: 8010-1308) (Agilent Technologies) was utilized for sample introduction, injecting 1 µL of sample into the front splitless inlet held at 230 °C. Helium served as the carrier gas at a constant flow rate of 1 mL/min. Chromatographic separations were performed on a DB-5 ms fused silica column (40 m × 250 µm I.D. × 0.25 µm film thickness) (Agilent Technologies, USA). The oven temperature was initially maintained at 45 °C, ramped to 120 °C at 15 °C min⁻^1^, then to 200 °C at 10 °C min⁻^1^, followed by a ramp to 280 °C at 5 °C min⁻^1^, and finally increased to 320 °C at 10 °C min⁻^1^, where it was held to complete a total runtime of approximately 37 min. The MSD transfer line temperature was set at 320 °C. The collision cell was supplied with helium at a flow rate of 2.25 mL/min.

A magnetic stirrer (Model 10MLH, REMI India) was used for sample preparation. Uniform coatings with a thickness of 70 μm were applied at room temperature using an automated film applicator (Elcometer 4340). Centrifugation was performed using an Eppendorf 5804R centrifuge. For the extraction of 21 organochlorine pesticides from the water matrix, a REMI CIS-18 Plus shaking incubator was employed. The basis for identifying individual OCPs has been described in Table S10.

FESEM (Carl Zeiss, USA; Model: SIGMA equipped with GEMINI column), EDX (Bruker, Germany; Model: Nano XFlash Detector), and FT-IR (Shimadzu Corporation, Japan).

### Statistical analysis

OriginPro 2025 (OriginLab Corporation, USA) software was utilized for the analysis of the data. The results in this study were represented as mean ± SE (standard error). The RSD value of multiple replicates was less than 10%. The blank studies were performed for each experiment during GC–MS/MS analysis. Reproducibility for concentration-dependent studies was assessed over the range of 100–900 ng/mL, with multiple replicate measurements. A linear fitting plot was generated from the calibration curve, and the linear regression coefficient was calculated in Origin.

### Synthesis of coating materials

**Fig. 1:**
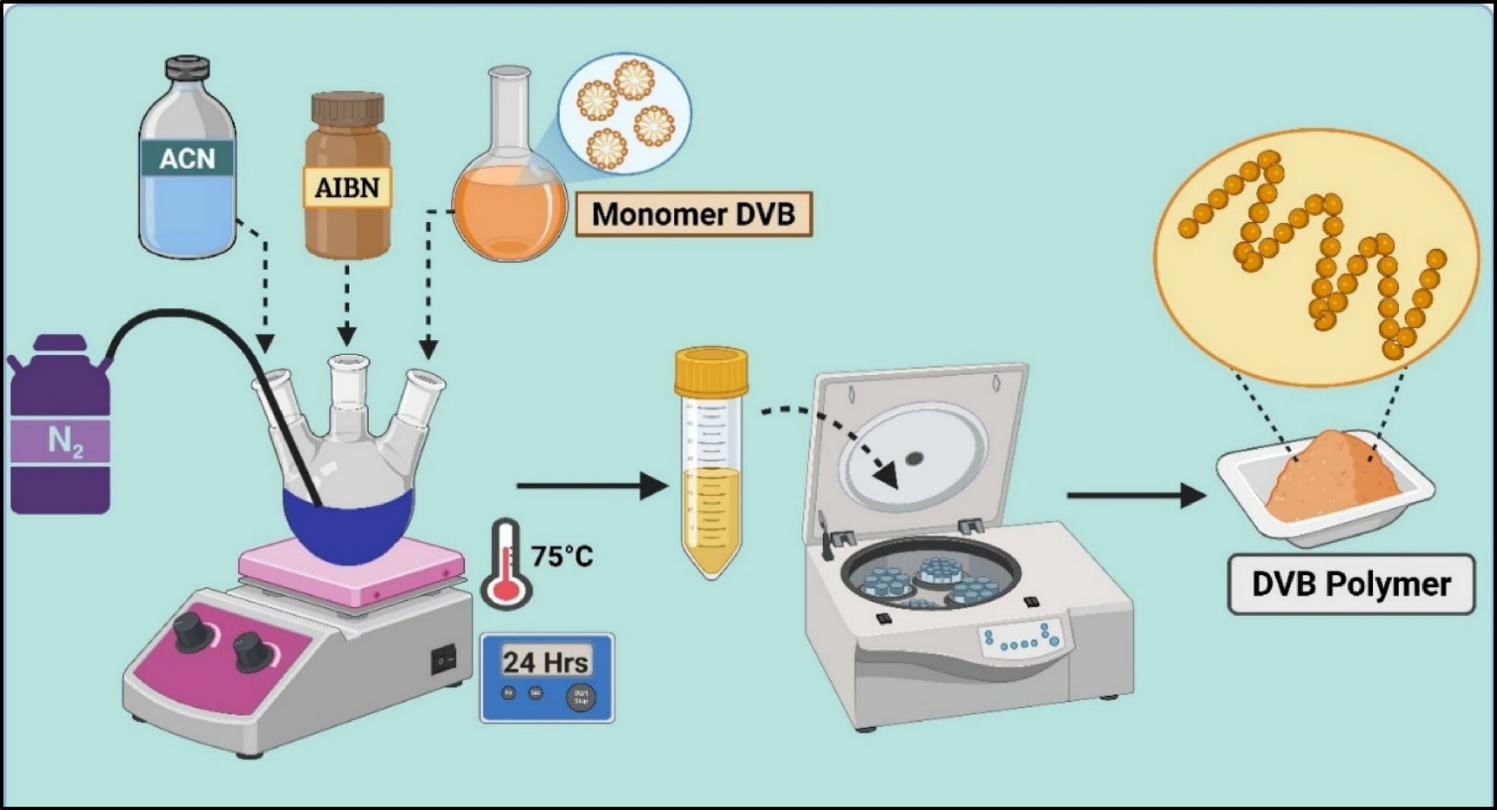
(**a**) Precipitation polymerization process for the synthesis of DVB particles.

Divinylbenzene (DVB) particles were synthesized (Fig. 1) by the precipitation polymerization process following the synthesis protocol of our previous study^42^. The obtained DVB particles of approximately 1–5 µm in size.

**Fig. 2:**
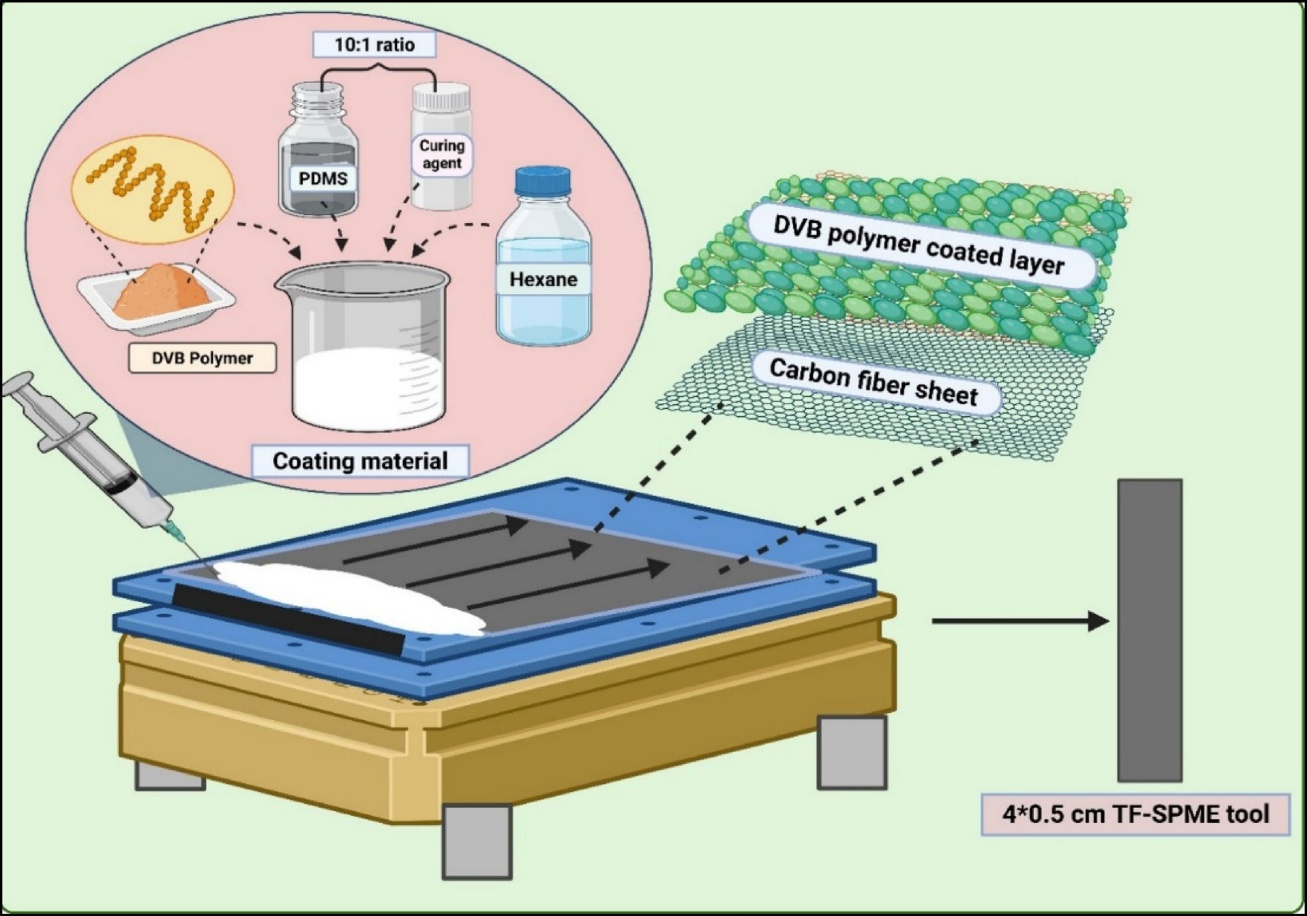
Preparation of coating recipe by homogenous mixing of the polymer and sorbent particles and Fabrication of carbon fiber-based TF-SPME patch with the utilization of film applicator.

A DVB/PDMS coating composition was prepared by adding 5 g of synthesized DVB polymers, 17 mL of Sylgard 186 elastomer, 1.7 mL of curing agent, and 40 mL of hexane to a beaker. The coating material was then placed on a magnetic stirrer for 24 h to obtain a homogeneous coating composition (Fig. 2).

### Fabrication of paper-based TF-SPME

The carbon fiber sheet was pre-cleaned with a combination of equal ratios of acetonitrile, isopropyl alcohol, and methanol before coating. Divinylbenzene/Polydimethylsiloxane (DVB/PDMS) coating was loaded into a syringe and applied evenly over the carbon sheet using the Elcometer 4340 automatic film applicator. The coating materials were applied to both the top and bottom surfaces of the sheet to achieve a thickness of approximately 70 µm at each side of the patch. The coating thickness was measured using an Elcometer Ultrasonic Precision Thickness Gauge equipped with a high-resolution probe for the measurement of the coating thickness of the carbon fiber sheet. Multiple measurements across the coated area of the patches confirmed an average coating thickness of 71 µm, with minimal variation of ± 1 µm. The coated carbon fiber sheets were kept in a hot oven with nitrogen for 12 h to remove any impurities. The sheet was then cut into a number of patches of dimensions 4 × 0.5 cm (Fig. 2), identical to the size of the commercially available patch marketed by MARKES International USA.

### Sample preparation method to extract 21 organochlorine pesticides from the water

The main stock solution was prepared containing 100 ppm of a mixture of 21 organochlorine pesticides in equal ratios of toluene and hexane. A mixture of toluene and hexane was used as the dilution solvent, as the EPA 8081 standard containing 21 organochlorine pesticides (Sigma–Aldrich) was originally supplied in the same solvent system, ensuring solvent compatibility during serial dilutions. It was stirred thoroughly using a magnetic stirrer to ensure homogeneity. The extraction was performed at different concentrations, containing a stock of 21 OCPs at levels ranging from 100 to 900 ng/mL in vials. To optimize the method of extracting the pesticides using our developed microextraction patch, we studied various analytical parameters including the concentrations, temperatures, extraction times, desorption times, and solvents. We compared the efficiency of the developed patches to that of commercially manufactured DVB/PDMS patches. Desorption for each experiment was achieved using 1 mL of acetonitrile to ensure that the analytes were entirely transferred from the patches to the solvent. Extraction and desorption were performed in a shaking incubator to ensure the same conditions for both procedures. The desorption was done using 1 mL ACN and was analyzed using GC–MS/MS to measure the 21 organochlorine pesticide residues.

## Results and discussion

**Fig. 3:**
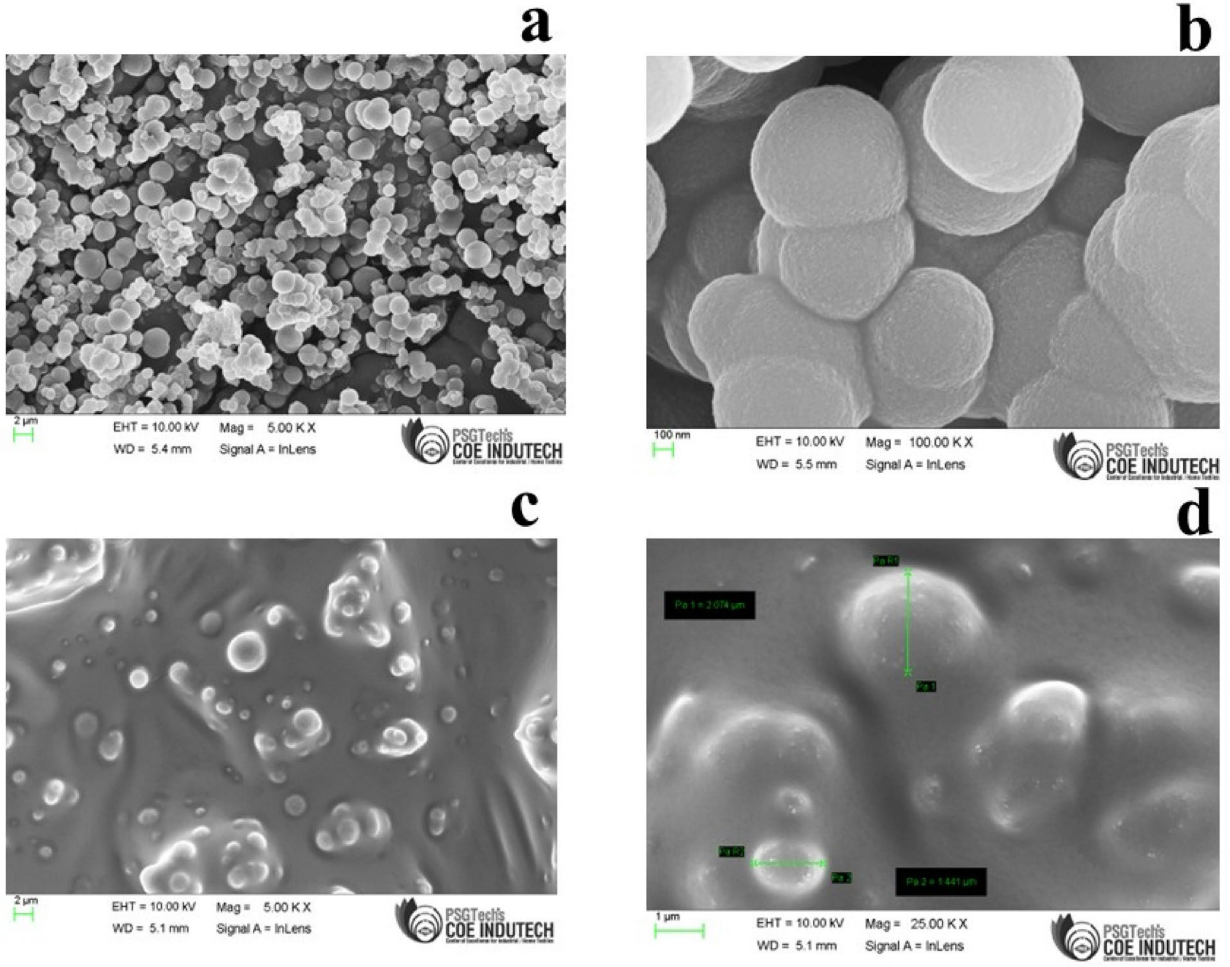
Field Emission Scanning Electron Microscopy (FE-SEM) images of the Divinylbenzene (DVB) particles (**a** and **b**); (**c** and **d**) FE-SEM image of coated carbon fiber-based TF-SPME patch.

The synthesized Divinylbenzene (DVB) particles were characterized using Field Emission Scanning Electron Microscopy (FE-SEM) and Energy Dispersive X-ray Spectroscopy (EDX) analysis. The FE-SEM images of the laboratory-made DVB particles were approximately 1–5 µm in diameter (Fig. 3a and b). The DVB/PDMS-coated patches confirmed the uniform distribution of the polymer particles on the coated carbon fiber-based substrate. Figure 3c and d show the intact morphological structure of the fabricated patches after coating on the carbon fiber substrate.

**Fig. 4:**
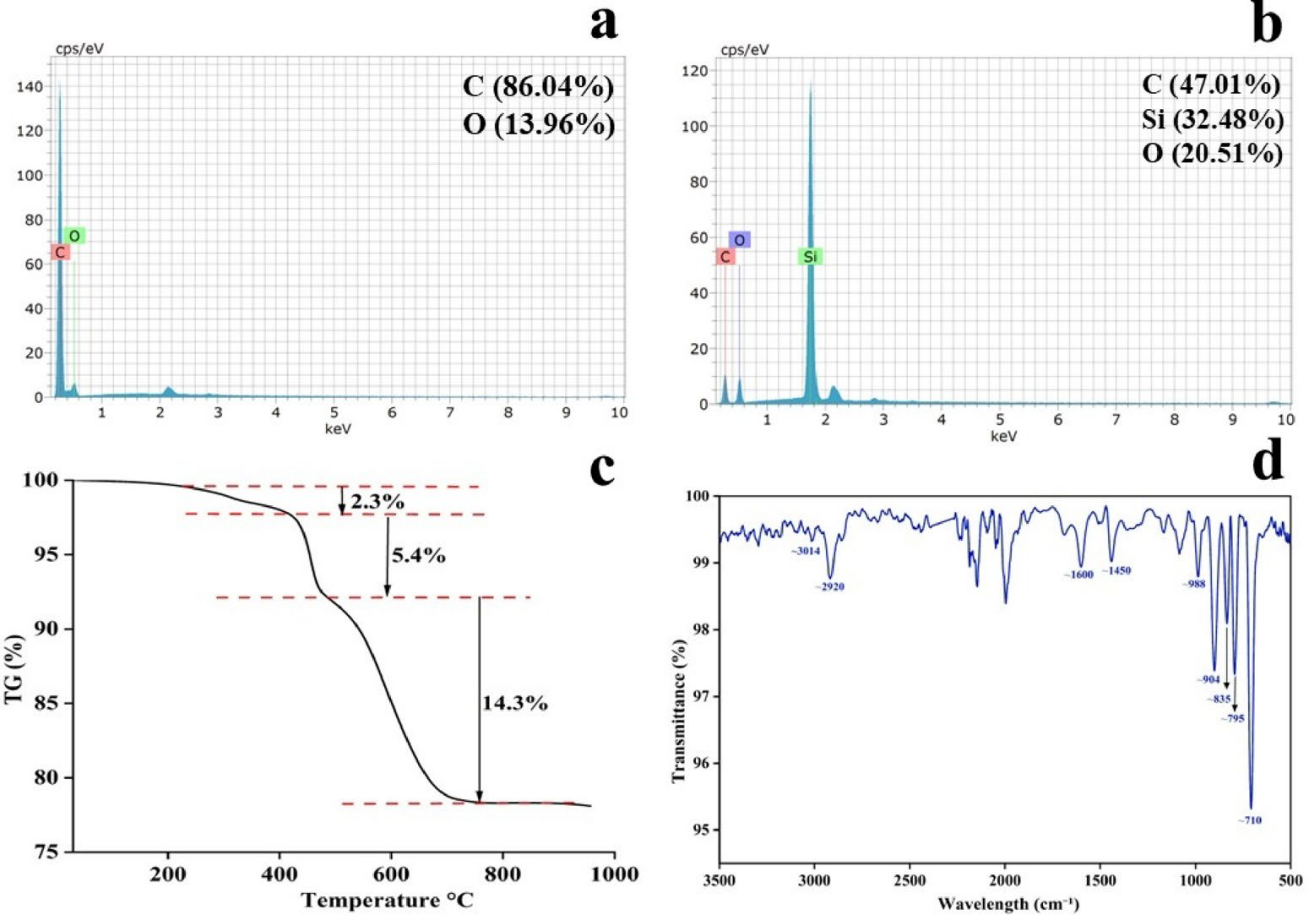
(**a**) Energy Dispersive X-ray spectrometry (EDX) of synthesized DVB particles and (**b**) DVB/PDMS coated carbon fiber-based TF-SPME patches: (**c**) Thermal stability of DVB/PDMS TF-SPME patch by Thermogravimetric analysis (TGA). (**d**) Fourier Transform Infrared Spectra (FT-IR) of synthesized DVB particles.

The elemental compositions of the DVB sorbent particles and DVB-coated carbon fiber sheet were investigated by energy dispersive X-ray spectrometry (EDX) as shown in Fig. 4a and b, indicating that the DVB particles contained 89.14% carbon, 10.86% oxygen and the coated carbon fiber patch contained 61.61% carbon, 20.18% oxygen, and 18.20% silicon, respectively by atomic percentage. In addition, EDX results confirmed that the particles were mainly composed of carbon with trace amounts of oxygen due to surface oxidation or impurities (Fig. 4a and b).

The Thermogravimetric Analysis (TGA) results showed a two-stage decomposition profile for the DVB particles. The first and second weight losses were 2.3% and 5.4%, respectively, at 420–483 °C, corresponding to residual amounts of unreacted monomers, volatile organic material, or surface-functionalized groups present in the DVB matrix. With increasing temperature, a further 14.3% weight loss was observed between 700 °C and 748 °C, reflecting the major decomposition of the DVB polymer network. Within this temperature range, the bulk material (i.e., the DVB organic framework) degrades into volatile products, leaving a carbonaceous residue. The stability of the degraded mass above 748 °C during TGA analysis indicated that the residual mass was largely composed of carbon (char) or inorganic residues (Fig. 4c).

The Fourier Transform Infrared Spectra (FT-IR) spectrum of divinylbenzene (DVB) showed the characteristic absorption bands for both vinyl and aromatic groups. An absorption at 3014 cm⁻^1^ was attributed to = C–H stretching vibrations of both the aromatic and vinyl groups, while 2920 cm⁻^1^ was attributed to the aliphatic C–H stretching of the vinyl CH₂ group. The strong absorption at 1600 cm⁻^1^ was attributed to C = C stretching vibrations of the aromatic ring, and that at 1450 cm⁻^1^ was for the aromatic ring skeletal vibrations related to in-plane C = C deformation. In the low wavenumber region, strong bands at 988 cm⁻^1^ and 904 cm⁻^1^ were characteristic of vinyl group-related out-of-plane = C–H bending, the presence of vinyl substitution. Additionally, the peaks at 835 cm⁻^1^ and 795 cm⁻^1^ were attributed to aromatic C–H bond out-of-plane bending, with contributions from para-/vinyl substitution and ortho-/meta-substitution, respectively. Finally, the absorption at 710 cm⁻^1^ was related to the out-of-plane bending vibration of aromatic C–H bonds, typical of a monosubstituted benzene ring (Fig. 4d). Furthermore, the TGA data showed ~ 2.3% mass loss between 420 and 480 °C, indicating the presence of trace levels of unreacted monomers. The FT-IR spectra further confirmed the efficient polymerization of the synthesized DVB particles.

In addition, the Fourier Transform Infrared spectrum (FT-IR) of polydimethylsiloxane/divinylbenzene patch (DVB/PDMS) was investigated (fig S5).

**Fig. 5:**
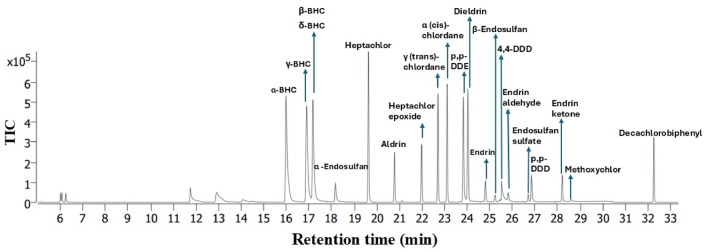
Chromatogram of 21 organochlorine pesticides after GC–MS/MS run.

The chromatogram from the GC–MS/MS study showed a clear separation of the 21 peaks associated with 21 organochlorine pesticides from water samples (Fig. 5).

### Optimization of various analytical parameters with carbon fiber patches

#### Effect of desorption time

**Fig. 6:**
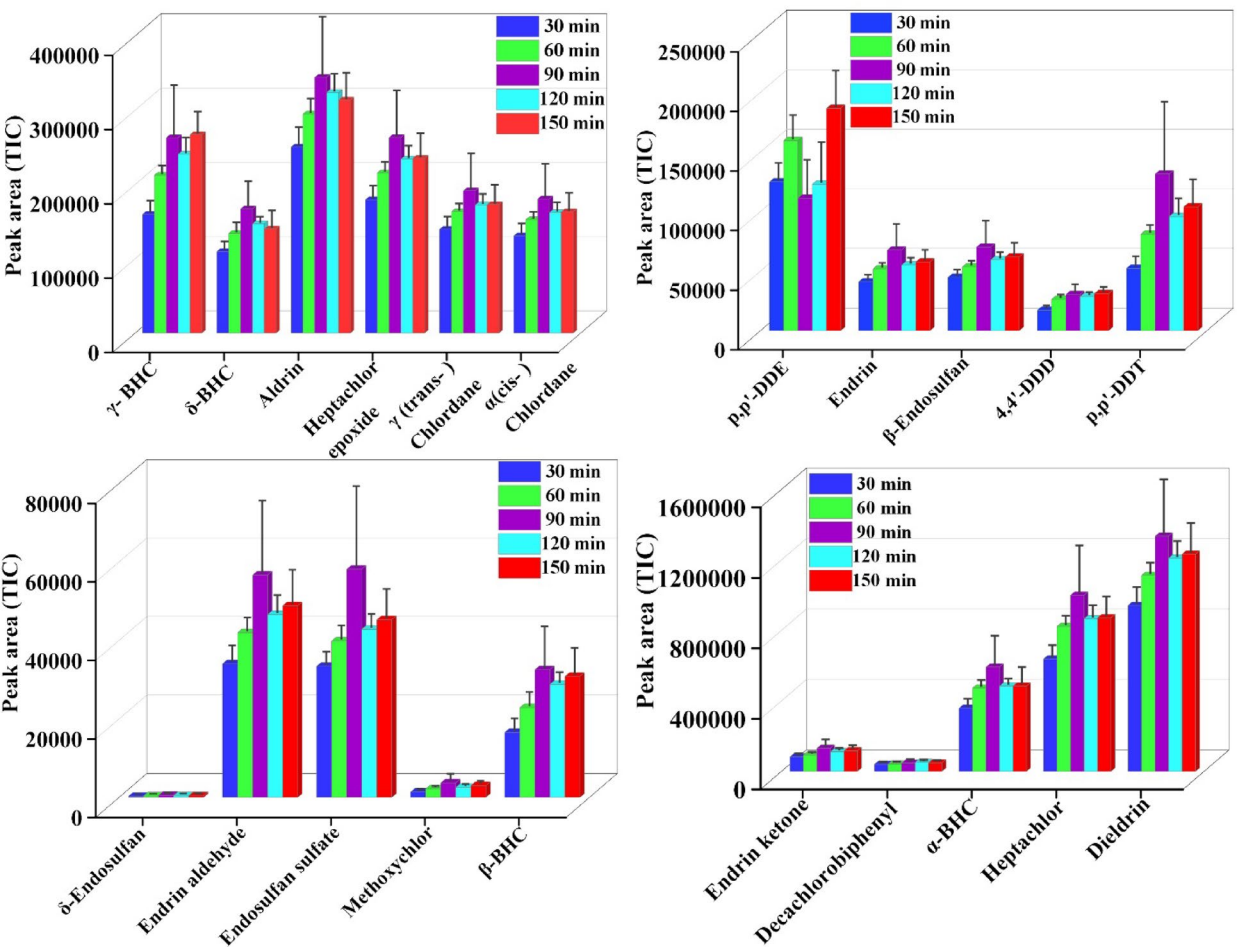
Effect of desorption Time: Evaluation of optimal desorption duration for efficient recovery of 21 OCPs.

Desorption time is a critical parameter for efficient recovery of the analytes from the coated carbon fiber to the desorbing solvent. Desorption of 21 organochlorine pesticides using DVB/PDMS-coated carbon fiber patch was investigated at different time intervals 30, 60, 90, 120, and 150 min. Desorption studies indicated that all compounds achieved effective release of extracted analytes within 90 min, indicating 90 min as the optimum desorption time. At 90 min, sufficient interaction time between the DVB/PDMS coating and the desorbing solvent was achieved, allowing most analytes to diffuse completely from the sorbent phase into the solvent. This duration allowed most of the compounds to reach desorption equilibrium, leading to efficient recovery. Extending the desorption time beyond 90 min did not significantly enhance the peak area, possibly because equilibrium had already been attained, and further solvent contact may have promoted re-adsorption of certain analytes back into the coating or even slight degradation during prolonged exposure. Therefore, 90 min was considered the optimal time for this study, with the phenomenon of maximum analyte release and prevention of secondary losses (Fig. 6 & Table S1).

#### Effect of extraction time

**Fig. 7:**
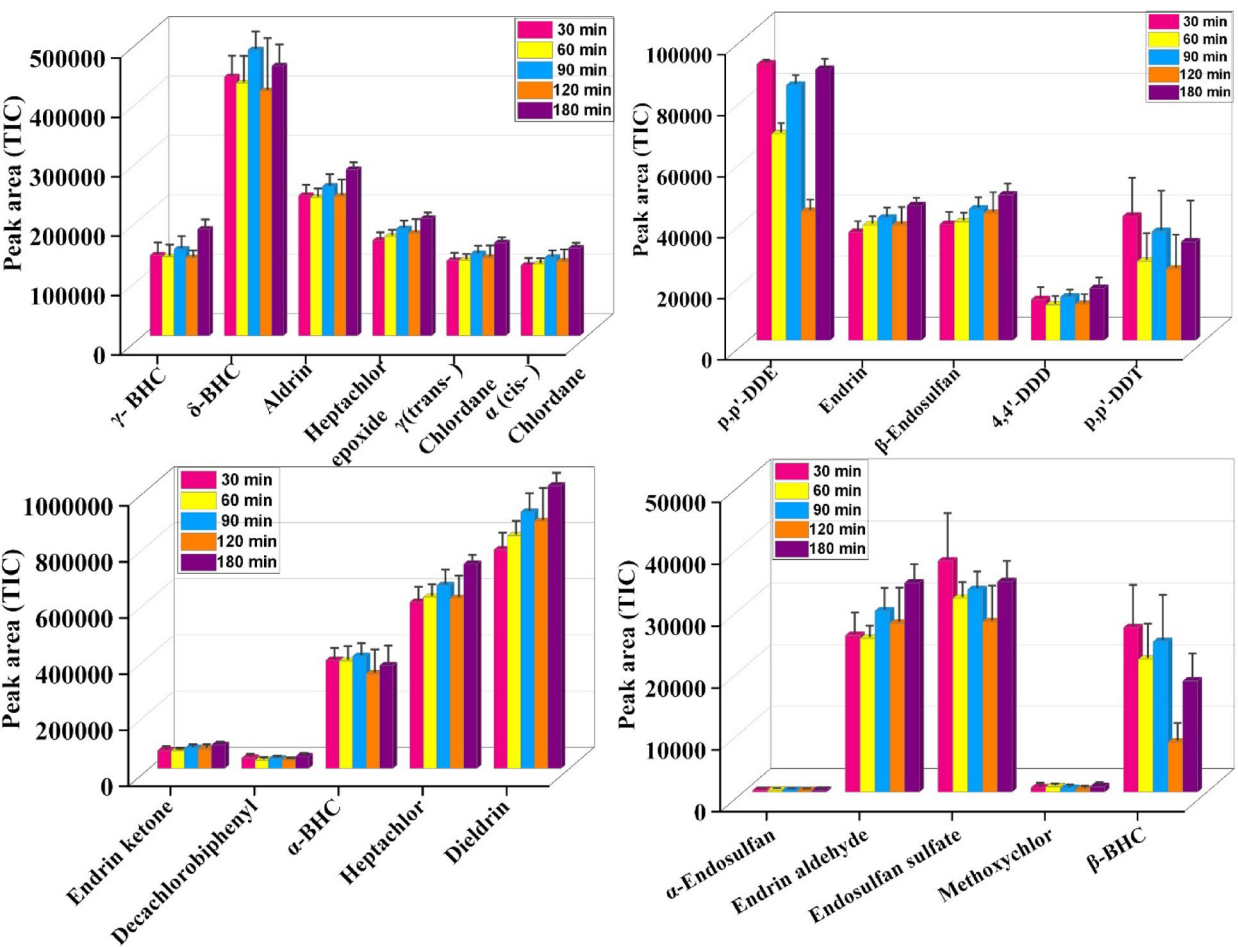
Effect of Extraction Time on the amount of 21 OCPs adsorbed by the patch.

Extraction time is a crucial parameter for determining the overall extraction efficiency of organochlorine pesticide residues using DVB/PDMS-coated carbon fiber patches. The peak areas for the different pesticides varied with extraction time (30, 60, 90, 120, and 180 min), indicating different levels of interaction between the analytes and the coating phase. A gradual increase in the peak area was observed with some pesticides, such as aldrin, dieldrin, and heptachlor, as the extraction efficiency continued to rise up to 180 min. Dieldrin and heptachlor showed substantial increases in peak area, indicating a high hydrophobicity and π-π interactions with the DVB moieties of the coated patch. Due to the high log *P* values and aromatic ring structure, these compounds efficiently partition into the polymer matrix. Some of the compounds, including endrin ketone, decachlorobiphenyl, and β-BHC (benzene hexachloride), showed relatively consistent or only slight increases in peak areas beyond 90–120 min, suggesting that equilibrium may have been attained earlier or that its interaction with the sorbent is relatively weaker. These trends indicated that specific physicochemical properties (ring structure, halogenation, and log *P* values) of the compounds govern the adsorption kinetics. With compounds such as α-BHC, γ-BHC, and methoxychlor, moderate extraction was observed, with peak area increasing gradually over 90 min, then plateauing or declining at longer times. The longer extraction times were favorable for some of the pesticides; however, most of the compounds were recovered or reached its highest extraction efficiency at 90 min. Considering all 21 OCPs, 180 min was chosen as the optimal time for the full recovery of all 21 organochlorine pesticides (Fig. 7 & Table S2).

#### Solvent desorption profile

**Fig. 8:**
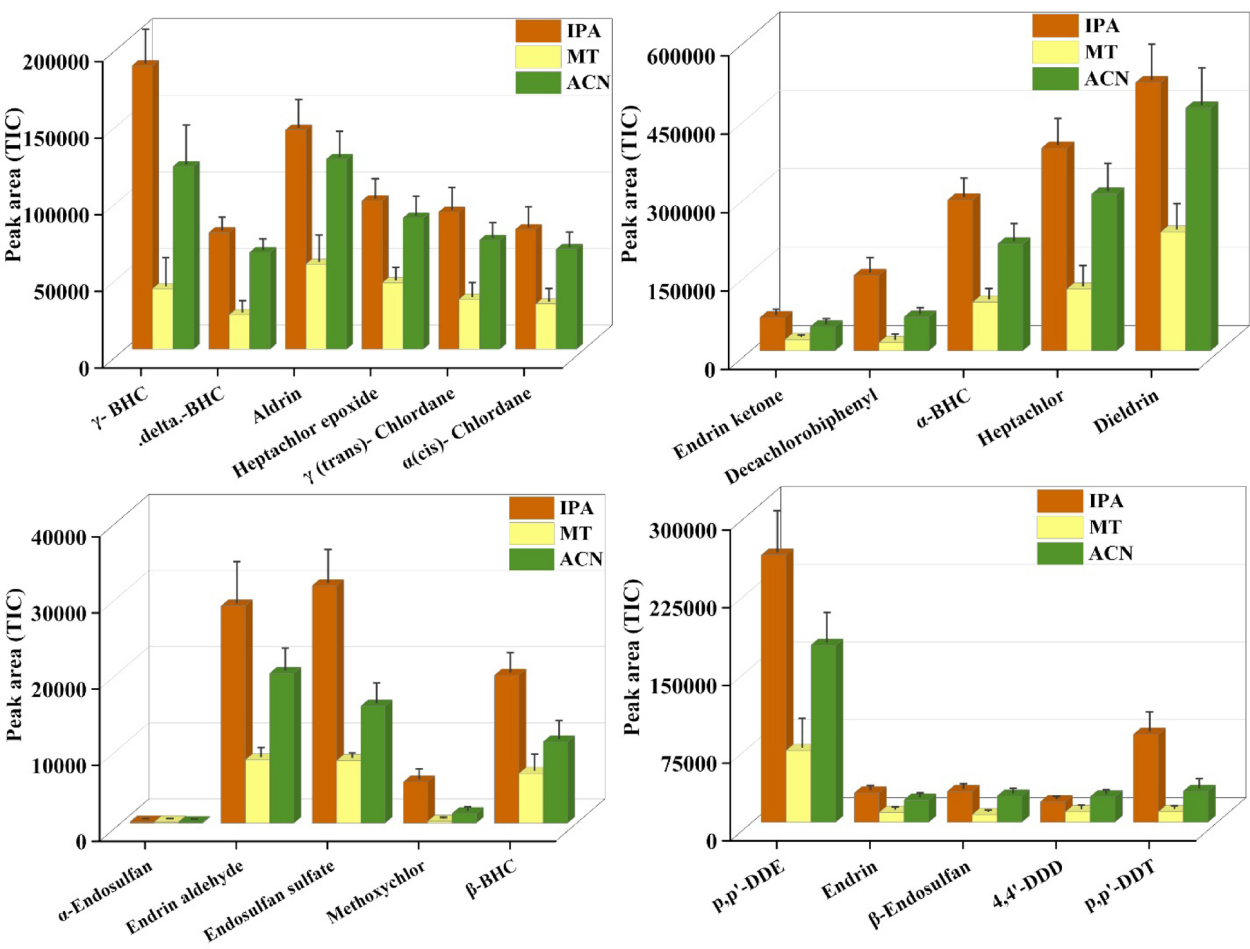
Effect of Desorption Solvent: Comparison of different solvents used for desorbing 21 OCPs from the patch.

The selection of desorption solvent plays a crucial role, as it directly influences the recovery percentage of the analytes from the sorbent phase. In the present study, three solvents, Isopropanol (IPA), Methanol (MT), and Acetonitrile (ACN), were evaluated for its desorption efficiency for 21 organochlorine pesticides. The efficiency of the solvents for desorbing analytes was compared after GC–MS/MS runs. Isopropanol (IPA) showed good desorption efficiency. The graph (Fig. 8) indicates a good interaction of IPA with semi-polar and non-polar analytes, which could be due to its intermediate polarity and its ability to disrupt the analyte-DVB/PDMS sorbent matrix interaction. ACN solvent did not outperform IPA in most cases, and it produced larger peak areas than methanol for several compounds, including dieldrin, heptachlor, and p,p-DDT. This indicated that ACN could serve as an alternative to IPA. Methanol (MT), however, exhibited low peak areas for almost all the pesticides studied, indicating poor desorption efficiency. The relatively low polarity of methanol compared to the other two solvents may be the primary reason for its ability to dissolve and extract non-polar organochlorine compounds from the DVB/PDMS coating. Overall, IPA proved to be the most effective desorption solvent, with consistent desorption of 21 OCPs. The results confirmed the importance of solvent selection in optimizing the SPME-based extraction procedure, particularly for semi-volatile and hydrophobic pollutants like organochlorine pesticides (Table S3). Although the solvent-profiling study demonstrated the highest desorption efficiency of IPA for several pesticide analytes, acetonitrile was used for all subsequent desorption experiments. We observed that ACN provided good compatibility with GC–MS/MS injection, low viscosity, and reduced background interference when handling a mixture of 21 pesticides in our study. Therefore, the desorption for this study was performed with 1 mL ACN solvent.

#### Effect of temperature

**Fig. 9:**
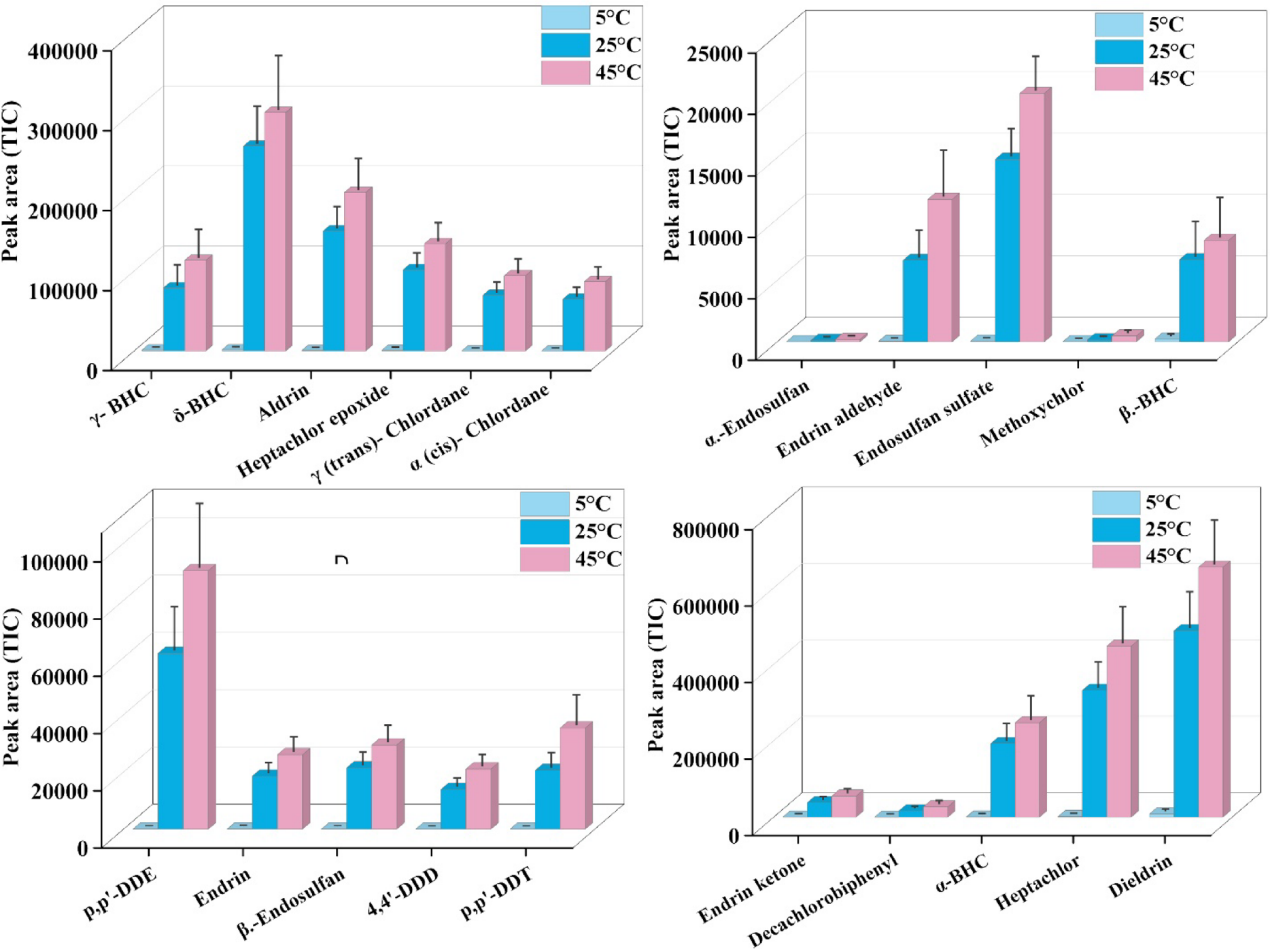
Effect of temperature: Assessment of temperature conditions on extraction efficiency.

The effect of extraction temperature was studied at 5 °C, 25 °C, and 45 °C under direct immersion. Among the temperatures 5, 25, and 45 °C, 45 °C yielded the largest peak areas, indicating an efficient extraction process. This improvement is due to several factors; increased temperature raises the diffusion coefficients of analytes in the aqueous phase, thereby allowing faster migration to the sorbent surface. Increased temperature reduces the viscosity of the solution and reduces the aqueous boundary layer thickness at the sorbent–solvent surface, thereby allowing faster mass transfer. The moderate increase in temperature provides sufficient thermal energy to overcome analyte–water interactions, allowing its partitioning to the DVB/PDMS-coated carbon fiber phase. In contrast, low temperatures (5 and 25 °C) limit diffusion and reduce analyte transfer, thereby lowering extraction efficiency. At 45 °C, no volatilization or degradation of the analytes was observed, suggesting that the temperature provides an optimal balance between improved mass transfer kinetics and analyte stability. Therefore, 45 °C was determined as the optimal temperature for efficient recovery of pesticides (Fig. 9 & Table S4). The extraction was not performed above 45 °C, as high temperatures increase the kinetic energy and weaken the intermolecular forces between the sorbent materials and OPCs. This can reduce the extraction efficiency of the patches.

In addition, the influence of pH and ionic strength on the extraction performance was also systematically evaluated (Fig S2 & Fig S3), providing a detailed overview of how these parameters affect the efficiency of the developed DVB/PDMS-coated TF-SPME patch.

#### Comparative study for the extraction of pesticides from carbon fiber-coated patches and commercially available TF-SPME patches

**Fig. 10:**
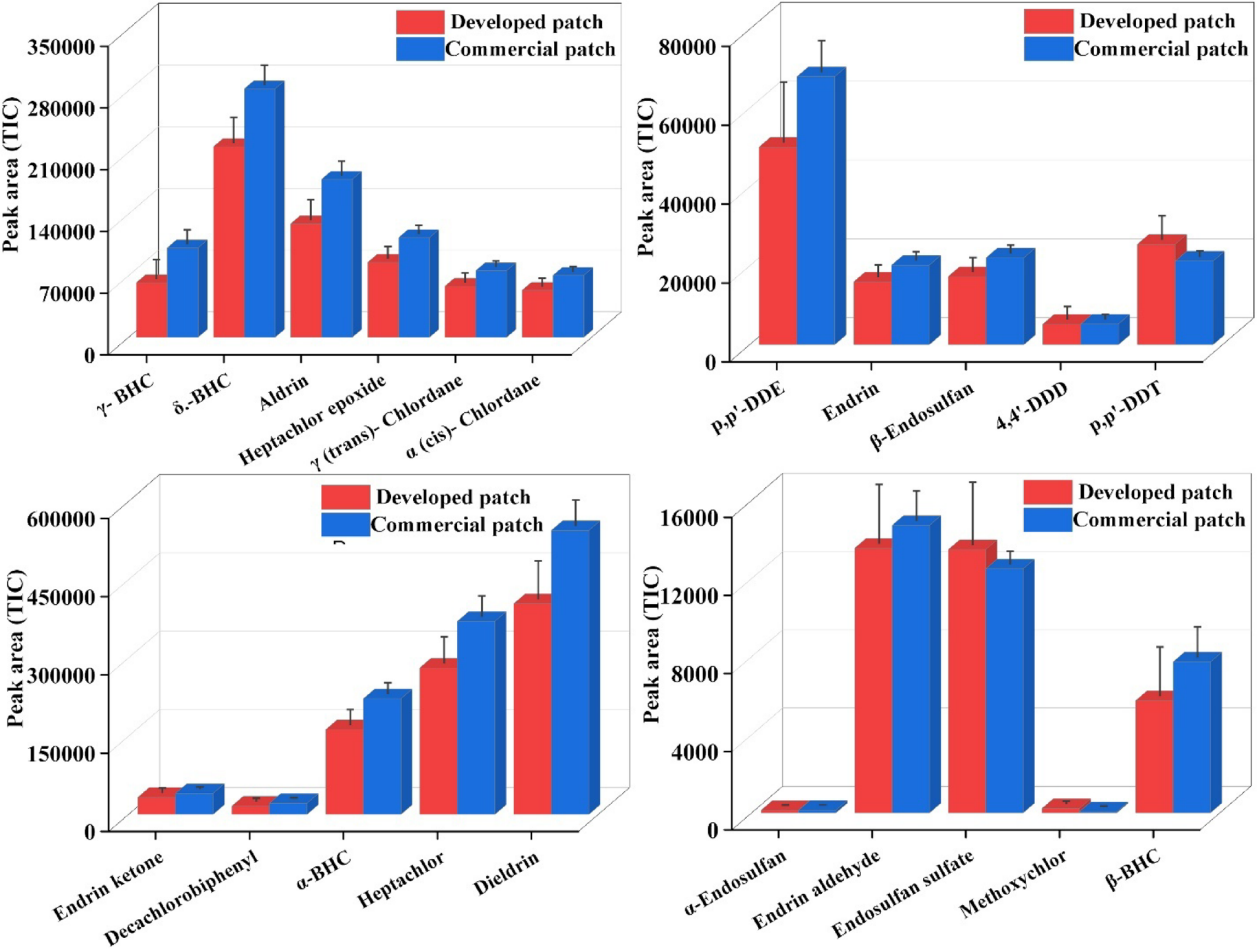
Comparative study with commercial DVB/PDMS-coated patches and carbon fiber-coated DVB/PDMS patches.

A comparative evaluation of the synthesized DVB/PDMS-coated carbon fiber patch with a commercially available patch revealed that the extraction efficiency for the majority of pesticides was comparable using both patches (Fig. 10). Endosulfan sulfate and p,p′-DDT showed superior extraction performance by the fabricated patches. This could be due to the increased hydrophobic interaction and strong affinity of the high-molecular-weight, non-polar molecules with the DVB/PDMS-coated carbon fiber surface, and it would possess a greater number of retention sites and stronger partitioning compared to the commercial patch. For the remaining analytes, although the synthesized patch showed a slightly lower extraction efficiency than the commercial one, the overall extraction was satisfactory. Significantly, our designed patches have a low production cost, and hence it is a cost-effective and readily available alternative for commercially sourced patches in the regular monitoring of organochlorine pesticides (Table S5). Furthermore, we reviewed the reported literature and evaluated our developed technique with previously established traditional extraction methods for the extraction of OCPs (Table S6) .

#### Concentration variation study

**Fig. 11:**
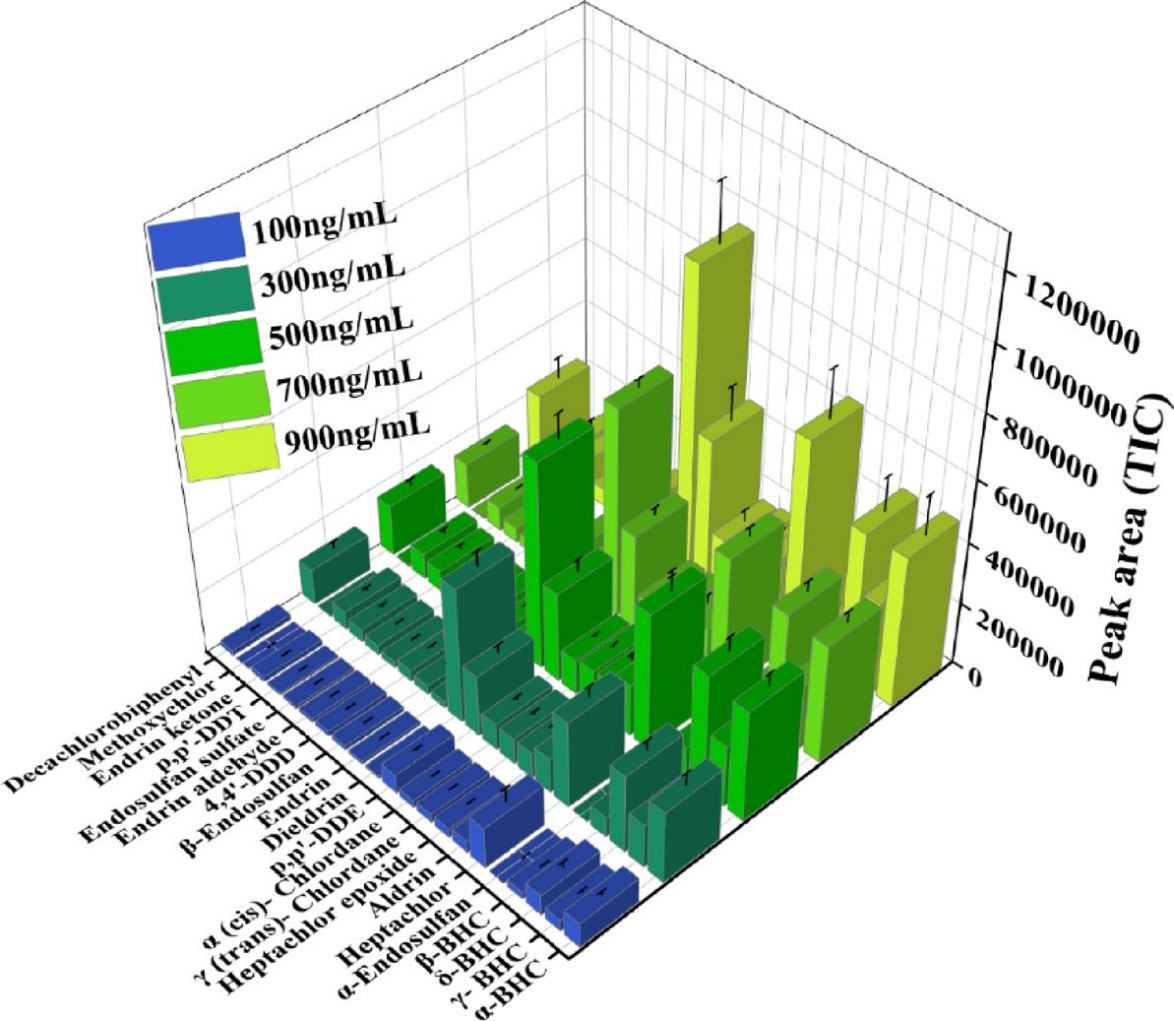
Extraction of 21 organochlorine pesticides from the water matrix. by carbon fiber-coated microextraction patches.

To assess the efficiency of extraction at different concentrations, the carbon fiber-based TF-SPME patch coated with DVB/PDMS was used to extract a wide range of organochlorine pesticides from water samples at five different concentrations: 100, 300, 500, 700, and 900 ng/mL, as depicted in Fig. 11. The 3D concentration variation plot illustrated the peak area responses of the 21 OCPs across five concentration levels (100, 300, 500, 700, and 900 ng/mL). Each bar corresponds to the peak area of an individual pesticide at a given concentration. The bar graph showed a distinct and steady increase with rising analyte concentrations, establishing the linearity and efficiency of patches for the extraction of the pesticides.

**Fig. 12:**
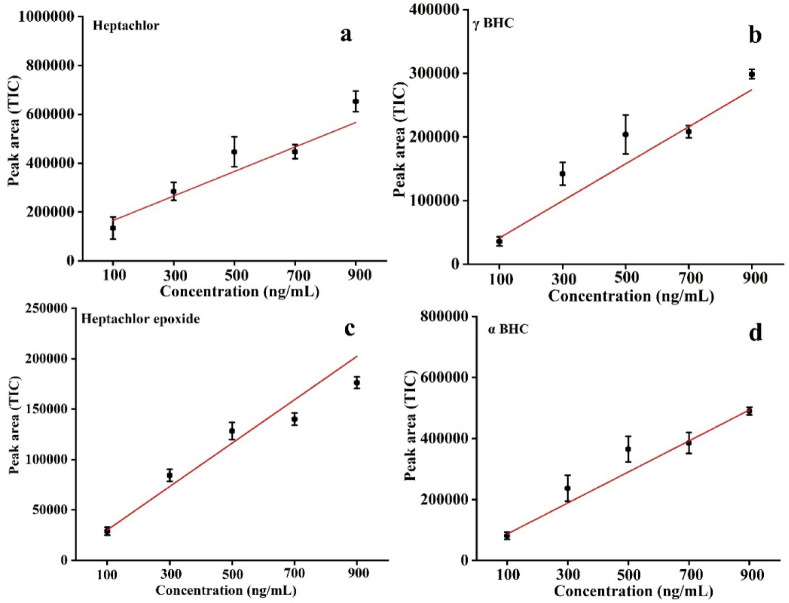
Calibration curve of (**a**) Heptachlor, (**b**) γ-BHC, (**c**) Heptachlor epoxide, (**d**) α-BHC.

The OCPs demonstrated a significant increase in peak areas, providing efficient interaction between the DVB/PDMS-coated patch and both polar and non-polar OCPs. This is due to the unique mixed-polarity nature of the DVB (moderately polar) and PDMS (non-polar) coating, which facilitates effective extraction through π–π interactions, van der Waals forces, and hydrogen bonding. Moreover, the extraction signal increased notably at high (700 and 900 ng/mL). These findings demonstrated the ability of the patches to generate calibration curves for quantifying unknown concentrations of multiple OCPs in water. The results confirmed that the developed patch offers a robust, sensitive method for the quantitative detection of OCPs, with good linearity across a wide concentration range, 100–900 ng/ml, with LOD of ~ 0.3–1.5 ng/mL for representative compounds. The R^2^ value for some analytes including endrin aldehyde was slightly lower compared to other analytes. This behavior is commonly observed for certain OCPs due to the inherent volatility, instability, and adsorption tendency of the compounds during sample preparation and dilution processes. The validation of the calibration curve was performed using the standard addition calibration approach^43^. The limit of detection (LOD) and limit of quantification (LOQ) were calculated using the standard deviation (σ) and the slope of the calibration curve, using the equations LOD = 3σ/slope and LOQ = 10σ/slope (Fig. 12, Fig. S1a–q and Table 1).

**Table 1:** Accuracy and LOD after spiking 21 OCPs into the water matrix.

Sample	Calibration equation	R^2^	LOD (ng/mL)	LOQ (ng/mL)
δ-BHC	Y = 15442.09874X − 30414.4524	0.93	1.2	3.6
γ-BHC	Y = 58195.86215X + 16753.85736	0.95	1	3.03
α-BHC	Y = 101728.32915X − 14854.2161	0.99	0.6	1.8
Decachlorobiphenyl	Y = 4622.14278X − 29512.9879	0.95	0.5	1.5
Methoxychlor	Y = 621.80179X + 266.54761	0.95	1.4	4.2
Endrin ketone	Y = 20578.26558X + 3510.57502	0.98	0.6	2.02
4,4′-DDD	Y = 18765.04758X − 1640.26856	0.98	0.8	2.6
p,p′-DDE	Y = 30938.53083X + 28168.55874	0.96	0.5	1.7
Endosulfan sulfate	Y = 51782.2251X + 14179.2225	0.95	0.9	2.9
Endrin aldehyde	Y = 69155.5084X + 5834.47931	0.9	1.5	4.7
α-Endosulfan	Y = 48.37861X − 11.4131	0.91	0.9	3.1
p,p′-DDT	Y = 13657.74107X − 6174.34279	0.98	0.8	2.8
β-Endosulfan	Y = 10448.2579X + 4657.86776	0.97	0.6	1.9
Aldrin	Y = 45562.36833X + 4250.10058	0.97	0.7	2.3
Heptachlor	Y = 100319.52726X + 64958.81	0.93	1.6	5
Dieldrin	Y = 234852.42758X + 228106.65	0.96	0.3	1.05
α-(cis) Chlordane	Y = 41577.4915X − 16340.74621	0.96	0.6	1.8
γ-(trans) Chlordane	Y = 42267.3587X − 13663.35828	0.95	0.5	1.9
Heptachlor epoxide	Y = 43063.28403X + 12852.92018	0.96	0.5	1.6
Endrin	Y = 7275.77311X + 3233.5526	0.96	1.1	3.5
β-BHC	Y = 28052.42917X + 4175.10868	0.93	0.7	2.4

#### Assessment of Greenness score of the technique using AGREE, Complex MoGAPI, and BAGI Metrics

To assess the environmental sustainability, the proposed method was analyzed with several green analytical tools to quantify the eco-friendliness. Analytical GREEnness Metric (AGREE)^44^, Complex Modified Green Analytical Procedure Index (ComplexMoGAPI)^45^, and Blue Applicability Index (BAGI)^46^. The Click Analytical Chemistry Index (CACI) (Fig S4a & Table S7), The Carbon Footprint Reduction Index (CaFRI) (Fig S4b & Table S8) and The Analytical Green Star Area (AGSA) (Fig S4c & Table S9).

**Fig. 13:**
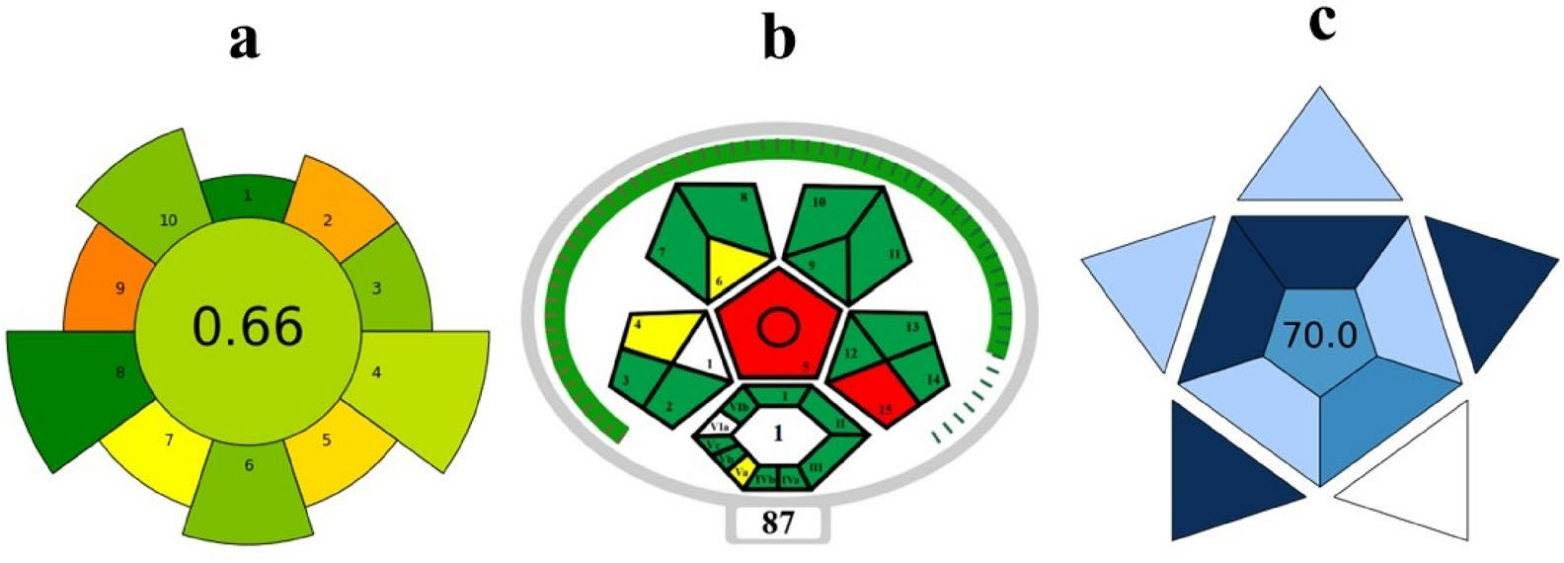
Green evaluation score by (**a**) AGREE (**b**) ComplexMoGAPI (**c**) BAGI.

The AGREE score of 0.66 (Fig. 13a). This score indicates that the suggested technique includes the use of a reduced amount of solvent (1 mL of ACN for desorption) compared to traditional solid-phase extraction (SPE) and liquid–liquid extraction (LLE) methods while maintaining the performance of the method. The carbon fiber-based thin-film solid-phase microextraction (TF-SPME) patch is characterized by its portability. Furthermore, this technique is associated with less waste generation, lower energy consumption, and efficient recovery of pollutants, thereby making it a sustainable and environmentally friendly technique. The AGREE assessment score emphasized the relation between the analytical performance and the environmental impacts of the developed method.

To evaluate the overall impacts of this method on human health and environmental factors, the Complex MoGAPI (Complex Modified Green Analytical Procedure Index) tool was used, as it was used in the pre-analytical sample preparation steps. The score was determined to be 87 after consideration of various analytical parameters, including reagent toxicity, instrumentation, energy efficiency, and waste disposal (Fig. 13b).

Following the greenness evaluation, the applicability of this method was studied using the Blue Applicability Grade Index (BAGI). The BAGI score was evaluated to be 70 (Fig. 13c), confirming the applicability and usability of the presented method for the analysis of organochlorine compounds in aqueous solutions according to sustainability^41^.

## Conclusion

The current research established the feasibility of using carbon fiber-based coated patches with a nanocomposite coating for the rapid extraction and quantification of organochlorine pesticides. The fabricated patches demonstrated the feasibility for utilizing it for routine purposes and requires minimal amount of solvent. The high surface area of the carbon fiber-coated patch improved the efficient extraction of target analytes, surpassing the recommended limits by environmental protection agencies. The method showed a limit of detection (LOD) of about 0.3–1.5 ng/mL, indicating its suitability for routine quality control testing. Although the current research used a small volume (1 mL) of solvent for analyte desorption from the patches, future research could involve directly connecting the patches to GC–MS/MS for organochlorine pesticide determination without solvent, thereby improving environmental sustainability compared to the approach outlined in this research. The major advantage of the current research is the economic feasibility of the analytical patch due to the use of low-cost carbon fiber as the coating substrate. The prepared patch could thus qualify as a viable substitute for commercial DVB/PDMS-coated TF-SPME patches (MARKES International USA), possibly lowering the manufacturing cost on a lab scale compared to its commercial equivalents. The patches were designed for single use only to eliminate the possibility of analyte carryover and requirement for conditioning. Due to low fabrication cost in laboratory scale, all the patches were utilized for a single time during the study. The results demonstrated the potential of the developed method as a sustainable and sensitive technique for monitoring priority pollutants in water bodies. The percentage recovery was not calculated in this study, as the TF-SPME technique is a microextraction method in which a very small quantity of analytes is adsorbed from the original sample matrix. Future studies may focus on estimating the recovery rate of this technique for comparison of this analytical sample preparation method with the traditional technique. The approach was checked with green analytical assessment tools like AGREE, Complex GAPI, and BAGI, and its compliance with the principles of green chemistry was assured. The results confirmed that the carbon fiber-based TF-SPME patch are an efficient and environmentally friendly tool for the analysis of environmental pollutants and its potential use in future analytical and environmental studies. The designed carbon fibre-based patches were not specific for any analyte present in the OCPs standard solution. However, we were able to get the resolved peak for individual analytes present in OCP standard. Additionally, in future works, the developed carbon fiber-based TF-SPME patch will be further validated using real environmental water samples to evaluate its performance under actual matrix conditions, and its integration with a portable mass analyzer can enable on-site analysis of toxic pollutants, thereby contributing to environmental protection and public health safety. This method can support local water-management authorities in rapid assessments, monitoring, and identification of contamination hotspots. The technique could be considered as a green practice due to the minimal use of organic solvents.

## Supplementary Information

Below is the link to the electronic supplementary material.

Supplementary Material 1

## Data Availability

The data of the current study were included in the manuscript and supplementary information sections. However, additional datasets could be obtained from the corresponding authors upon reasonable request.
