# Supplementary material for: Development of a carbon fiber-based microextraction sample preparation patch for the detection of 21 organochlorine pesticides from water
Source: Sci Rep. 2026 Jan 28;16:6543. doi: 10.1038/s41598-026-36604-0 (PMC12909302; doi:10.1038/s41598-026-36604-0)
Supplement: Supplementary file 1 — Supplementary Material 1 [file 41598_2026_36604_MOESM1_ESM.docx]

**Development of a carbon fiber-based microextraction sample preparation patch for the detection of** **21 organochlorine pesticides from water**

Harshika Poojary^a^ and Chiranjit Ghosh^a^ *

^a^Manipal Institute of Technology, Manipal Academy of Higher Education, Manipal, Karnataka, 576104, India.

*corresponding author: Chiranjit Ghosh

Email: [chiranjit.ghosh@manipal.edu](mailto:chiranjit.ghosh@manipal.edu)

This supplementary material provides the calibration curves of calibration plots for organochlorine pesticides (OCPs) by graphing concentration vs. peak area (TIC) for 100–900 ng/mL (Figures S1a–S1q) and provides the tables(S1-S5) containing the mean peak area values along with the corresponding standard errors, which were used for generating the error bar plots in the optimization studies. These datasets ensure transparency and reproducibility of the calibration and optimization experiments. It includes the effects of pH and salt concentration on extraction efficiency, and (table S6) which presents a comprehensive comparison of various extraction techniques used for determining organochlorine pesticides (OCPs) in water, the FT-IR spectra of the DVB/PDMS coated patch was analyzed (figS5). The greenness of the proposed method has also been critically assessed using CACI, CaFRI, and AGSA tools, and the specific parameters along with the corresponding values used for calculating the CACI score are provided in an accompanying (fig S4 & table S7-S9). Additionally, Base Peak and Fragment Ions (m/z) used for the identification of the 21 OCP have been mentioned in table S10.

Aldrin (a), α- endosulfan (b), δ-BHC (c), α-cis-chlordane (d), p,p′-DDE (e), and dieldrin (f) were observed to possess good linearity with high peak responses across the studied range. Decachlorobiphenyl (g), endrin ketone (h), methoxychlor (i), p,p′-DDT (j), endosulfan sulfate (k), and endrin aldehyde (l) were observed to possess linear trends, however, methoxychlor and p,p′-DDT exhibited relatively lower sensitivity. Endrin (m), β-endosulfan (n), 4,4′-DDD (o), γ-(trans) Chlordane (p), β-BHC (q) showed good concentration correlation and consistent reproducibility across replicates.

Satisfactory detector response and reproducibility of the calibration curves for all fifteen pesticides were obtained, ensuring that the established GC-MS method can be employed for reliable and accurate quantification of the OCPs within the test concentration range.


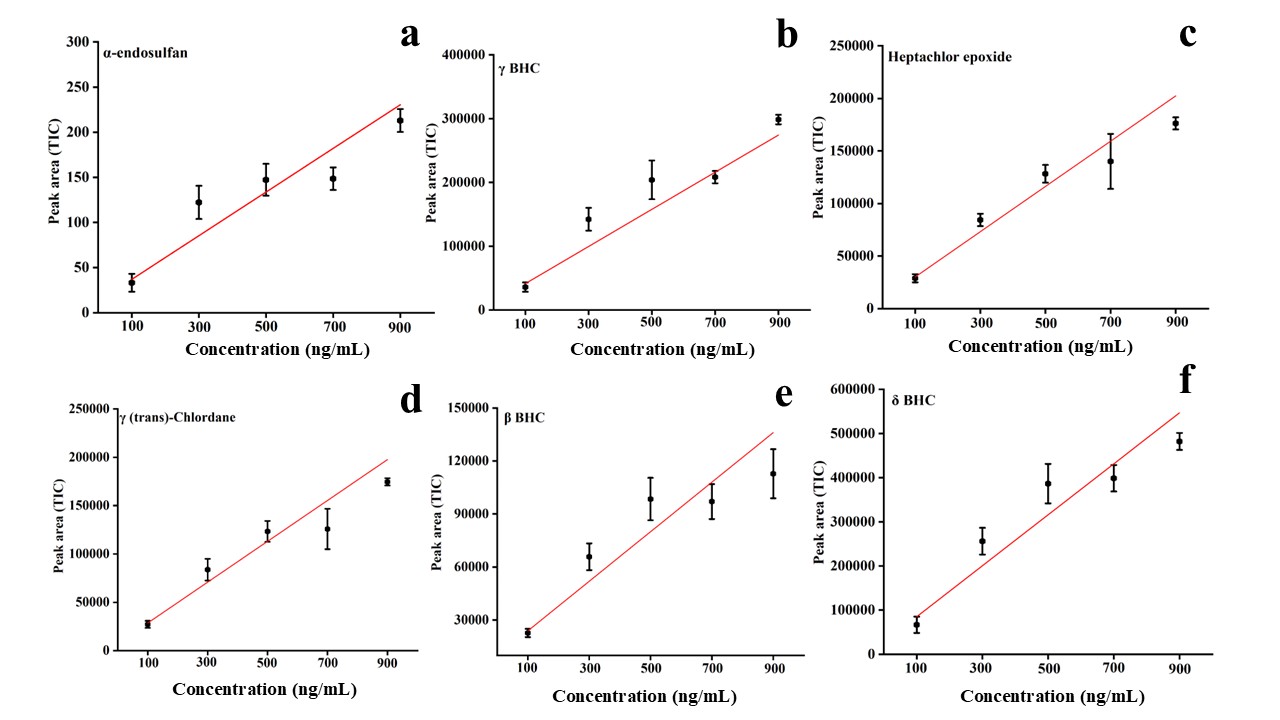

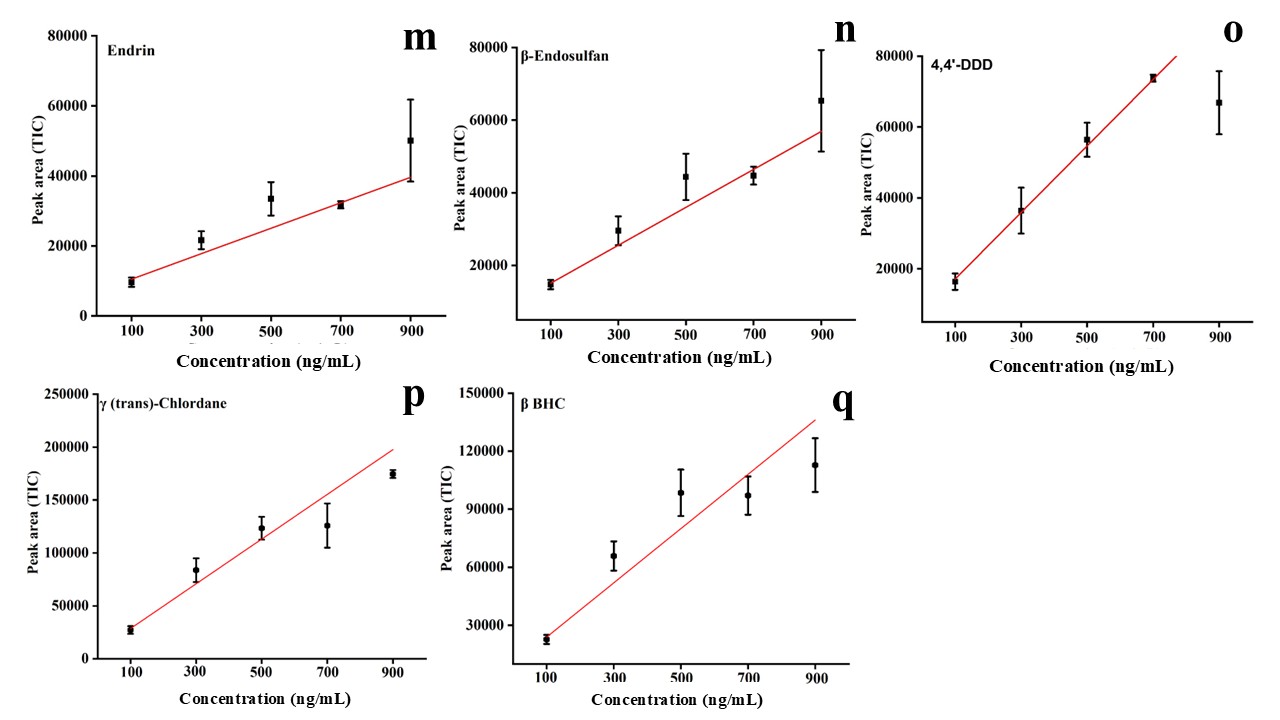


**Figure. S1**. Calibration curves of (a) Aldrin, (b) α- endosulfan, (c) δ-BHC, (d) α-cis-Chlordane, (e) p,p′-DDE, (f) Dieldrin, (g) Decachlorobiphenyl, (h) Endrin ketone, (i) Methoxychlor, (j) p,p′-DDT, (k) Endosulfan sulfate, (l) Endrin aldehyde, (m) Endrin, (n) β-Endosulfan, and (o) 4,4′-DDD, (p) γ-(trans) Chlordane, (q) β-BHC were obtained under a concentration range of 100–900 ng/mL.

The tables summarizes the optimization of desorption time (Table S1), extraction time (Table S2), solvent profiling (Table S3), temperature variation (Table S4), and comparative studies between developed patch and commercial patch (Table S5), For each pesticide the mean peak area obtained from GC-MS/MS data the five replicates was calculated and along with its corresponding standard errors. These mean peak area and standard errors were then further used for plotting error bar graph, where standard error as y error

**Table S1. Desorption time variation for 21 organochlorine pesticides with mean peak area and corresponding standard error.**

|  | **30 min** |  |  | **60 min** |  |  | **90 min** |  |  | **120 min** |  |  | **150 min** |  |
| --- | --- | --- | --- | --- | --- | --- | --- | --- | --- | --- | --- | --- | --- | --- |
| **Name of the pesticdes** | **Mean peak area** | **Standard error** |  | **Mean peak area** | **Standard error** |  | **Mean peak area** | **Standard error** |  | **Mean peak area** | **Standard error** |  | **Mean peak area** | **Standard error** |
| **gamma- BHC** | 160001.8 | 16264.47497 |  | 212600.4 | 10992.39802 |  | 263133.6 | 68542.97843 |  | 240860 | 20586.85911 |  | 267287.25 | 28862.57656 |
| **.delta.-BHC** | 109912.2 | 11682.82281 |  | 134291.8 | 12861.01598 |  | 167302 | 35255.3879 |  | 147080.8 | 7541.42853 |  | 140185 | 22757.34965 |
| **Aldrin** | 250542.4 | 24804.93894 |  | 294947 | 18805.15152 |  | 344425.8 | 79580.00457 |  | 323914.4 | 23204.13335 |  | 313772.25 | 34872.63826 |
| **Heptachlor epoxide** | 179598.8 | 16855.45905 |  | 215834.8 | 12787.61522 |  | 263383 | 61441.9252 |  | 234224.8 | 15898.65784 |  | 235320.5 | 31879.63875 |
| **gamma (trans)- Chlordane** | 139746.6 | 14962.40151 |  | 163613 | 8725.66475 |  | 191733.2 | 47910.76557 |  | 172444.6 | 12838.64504 |  | 172991.25 | 24809.65911 |
| **alpha (cis)- Chlordane** | 131138 | 14284.30613 |  | 152597.8 | 8863.83882 |  | 180712.6 | 45126.40233 |  | 162243.8 | 11896.76859 |  | 163389.75 | 23431.88263 |
| **p,p'-DDE** | 124945 | 14316.40071 |  | 159602 | 19847.05124 |  | 111378 | 30524.86218 |  | 122967 | 33755.01092 |  | 186742.25 | 30088.02553 |
| **Endrin** | 41116.8 | 4321.90157 |  | 51949.6 | 3384.11393 |  | 67690.4 | 20409.46925 |  | 55059.4 | 4699.16828 |  | 57774.75 | 8536.27634 |
| **.beta.-Endosulfan** | 44796.2 | 4921.84912 |  | 53555.6 | 3667.27551 |  | 70260 | 20383.45805 |  | 59521.4 | 4915.40515 |  | 61887 | 10346.38534 |
| **4,4'-DDD** | 17172.2 | 2396.61312 |  | 26319.6 | 2601.15191 |  | 30416.8 | 7069.22499 |  | 28670.4 | 2408.40172 |  | 31204.5 | 4154.59221 |
| **p,p'-DDT** | 52449.6 | 8622.74156 |  | 81090.2 | 6219.32147 |  | 131557.8 | 59202.04061 |  | 95956.2 | 13661.27975 |  | 104031 | 21570.87171 |
| **Endrin ketone** | 82653.2 | 9172.96146 |  | 93696.2 | 7779.18325 |  | 131599.8 | 40028.56871 |  | 111949.6 | 10710.61669 |  | 117061 | 20976.23379 |
| **Decachlorobiphenyl** | 40268.2 | 5107.98503 |  | 41562 | 4505.93622 |  | 46517 | 13861.16288 |  | 50916.2 | 6695.99992 |  | 48253.25 | 7247.54246 |
| **.alpha.-BHC** | 358472.2 | 44805.97263 |  | 472349.2 | 36684.66502 |  | 592338.8 | 167828.4156 |  | 484218.2 | 32593.15576 |  | 484725.25 | 97327.29297 |
| **Heptachlor** | 638208.8 | 68539.82858 |  | 823148.2 | 51175.67585 |  | 1000152.6 | 272071.4061 |  | 866317 | 66990.61914 |  | 872188 | 110904.8752 |
| **Dieldrin** | 942232.4 | 93659.89271 |  | 1112403 | 62070.18285 |  | 1334944.2 | 313204.4393 |  | 1209783.4 | 87268.67478 |  | 1233309.75 | 166215.0059 |
| **.alpha.-Endosulfan** | 307 | 43.55112 |  | 387.6 | 33.18825 |  | 550.2 | 158.09314 |  | 434.4 | 39.87305 |  | 439 | 89.69114 |
| **Endrin aldehyde** | 34069 | 4211.99498 |  | 41986.2 | 3327.74052 |  | 56675 | 18453.30254 |  | 46600.4 | 4480.82611 |  | 48869.75 | 8629.97552 |
| **Endosulfan sulfate** | 33434 | 3169.82427 |  | 39864.2 | 3491.56231 |  | 58147.8 | 20706.24519 |  | 42848.2 | 3447.59463 |  | 45288 | 7341.58456 |
| **Methoxychlor** | 1400.4 | 305.52195 |  | 2194 | 298.49154 |  | 3725.8 | 1832.71305 |  | 2565.2 | 386.702 |  | 3027.75 | 761.21628 |
| **.beta.-BHC** | 16606 | 3052.87 |  | 22884 | 3486.03 |  | 32545. | 10542.5 |  | 28814. | 2604.09 |  | 30871.2 | 6763.69 |

**Table S2. Extraction time variation for 21 organochlorine pesticides showing mean peak area and corresponding standard error.**

|  | **30 min** |  |  | **60 min** |  |  | **90 min** |  |  | **120 min** |  |  | **150 min** |  |
| --- | --- | --- | --- | --- | --- | --- | --- | --- | --- | --- | --- | --- | --- | --- |
| **Name of the pesticdes** | **Mean peak area** | **Standard error** |  | **Mean peak area** | **Standard error** |  | **Mean peak area** | **Standard error** |  | **Mean peak area** | **Standard error** |  | **Mean peak area** | **Standard error** |
| **γ- BHC** | 135700.25 | 18824.46264 |  | 132583 | 18375.30859 |  | 145446.75 | 19418.82734 |  | 130934 | 9996.14933 |  | 179241.8 | 13985.21093 |
| **δ.-BHC** | 436080 | 33043.84888 |  | 425247 | 43430.88869 |  | 481202 | 28633.52539 |  | 412250 | 86175.64065 |  | 454446.2 | 33329.22782 |
| **Aldrin** | 235838.25 | 15932.36383 |  | 232200.8 | 12906.20186 |  | 251344 | 18194.26377 |  | 234701 | 25818.45058 |  | 280160.8 | 9009.14186 |
| **Heptachlor epoxide** | 160512.25 | 10751.88012 |  | 166878.8 | 8799.66002 |  | 180045.5 | 11061.63448 |  | 172373.3333 | 21259.86579 |  | 197256 | 7718.25413 |
| **γ (trans)- Chlordane** | 126523.75 | 10545.39887 |  | 127040 | 8004.56798 |  | 137867.75 | 10861.01883 |  | 131348.6667 | 18047.55371 |  | 156512 | 6612.86379 |
| **p,p'-DDE** | 90612 | 831.82329 |  | 67629 | 2981.21911 |  | 83617 | 2718.98301 |  | 42312.66667 | 3293.43922 |  | 88843.4 | 2818.31204 |
| **Endrin** | 35417.75 | 3046.76906 |  | 37787.2 | 2265.11003 |  | 40101.25 | 2811.09191 |  | 37758.33333 | 5331.29032 |  | 44178.4 | 1962.35159 |
| **β.-Endosulfan** | 38048 | 3428.55453 |  | 38828.2 | 2427.87931 |  | 43079.5 | 3165.0562 |  | 41603.33333 | 6348.02408 |  | 47671.8 | 3116.86922 |
| **4,4'-DDD** | 13373.5 | 3453.17927 |  | 11577 | 2302.46249 |  | 14225.25 | 1851.52772 |  | 11793.66667 | 2694.93552 |  | 17079.2 | 2910.15913 |
| **p,p'-DDT** | 40676.25 | 11993.56272 |  | 25851.6 | 8635.31812 |  | 35652.25 | 12838.8178 |  | 23383 | 10658.33814 |  | 32294.6 | 12876.75132 |
| **Endrin ketone** | 64060.25 | 9445.29656 |  | 61830.2 | 4428.17741 |  | 71882 | 7651.5095 |  | 68925.33333 | 11019.47165 |  | 83365.6 | 6360.47146 |
| **Decachlorobiphenyl** | 37212.75 | 8720.48414 |  | 29341 | 5171.5305 |  | 34718 | 4222.36719 |  | 29782.66667 | 3469.90251 |  | 42762.4 | 6483.43868 |
| **α-BHC** | 385765.25 | 37840.9837 |  | 382697.6 | 47375.44617 |  | 400327.5 | 40446.31579 |  | 337639 | 80551.21699 |  | 367201.4 | 64136.17236 |
| **Heptachlor** | 592909.5 | 49324.78663 |  | 610611.2 | 39492.97165 |  | 652920.75 | 49894.92855 |  | 607299 | 74712.92478 |  | 729215.2 | 26262.12523 |
| **Dieldrin** | 780130.75 | 54083.18603 |  | 829149.2 | 47390.77054 |  | 914829.25 | 60721.29184 |  | 881616.6667 | 112642.705 |  | 1008607.4 | 39522.66247 |
| **α-Endosulfan** | 274.5 | 15.77709 |  | 291.8 | 29.07129 |  | 266.5 | 9.66523 |  | 234 | 54.55578 |  | 297.2 | 10.64143 |
| **Endrin aldehyde** | 25317.5 | 3395.12831 |  | 24822.2 | 1758.7886 |  | 29274.25 | 3408.77782 |  | 27285.33333 | 5479.06118 |  | 33830.2 | 2713.35059 |
| **Endosulfan sulfate** | 37363.25 | 7422.84188 |  | 31294.6 | 2317.29909 |  | 32746.25 | 2590.44364 |  | 27517.33333 | 5552.03417 |  | 34012.6 | 3040.31664 |
| **Methoxychlor** | 693.25 | 461.4402 |  | 848.6 | 228.93375 |  | 620.25 | 315.44846 |  | 388 | 324.52889 |  | 902.8 | 394.62532 |
| **β-BHC** | 26586.5 | 6622.14031 |  | 21449.6 | 5476.19311 |  | 24321.75 | 7241.50372 |  | 8086 | 2769.52541 |  | 17965.6 | 4124.16904 |
| **α (cis)- Chlordane** | 118583.25 | 9633.95101 |  | 120441.4 | 7359.88089 |  | 131424.5 | 9595.07795 |  | 125287.6667 | 17261.35849 |  | 147565.2 | 6113.80512 |

**Table S3. Solvent profiling with isopropanol (IPA), methanol, and acetonitrile (ACN) for desorption of 21 organochlorine pesticides showing mean peak area and corresponding standard error**

|  | **Isopropyl Alcohol** |  |  | **Methanol** |  |  | **Acetonitrile** |  |
| --- | --- | --- | --- | --- | --- | --- | --- | --- |
| **Name of the pesticdes** | **Mean peak area** | **Standard error** |  | **Mean peak area** | **Standard error** |  | **Mean peak area** | **Standard error** |
| **γ- BHC** | 185042 | 22065.27923 |  | 39542.75 | 18571.50909 |  | 119259.6 | 25190.81205 |
| **.delta.-BHC** | 76061.8 | 8442.39007 |  | 22487.25 | 7476.83009 |  | 63199.2 | 7025.09938 |
| **Aldrin** | 143017.6 | 18081.45612 |  | 55477.75 | 17211.86802 |  | 123933 | 16585.41437 |
| **Heptachlor epoxide** | 96957 | 12438.71206 |  | 43502.25 | 8184.62679 |  | 85614.4 | 12437.02639 |
| **γ (trans)- Chlordane** | 89493 | 14393.35896 |  | 32633.5 | 9115.27347 |  | 71152.2 | 9784.27241 |
| **p,p'-DDE** | 257942.8 | 39923.97531 |  | 69304.5 | 27956.10585 |  | 170972.8 | 28605.95305 |
| **Endrin** | 28344.8 | 4161.18524 |  | 9512.5 | 2222.96489 |  | 21467.4 | 3669.60789 |
| **β-Endosulfan** | 29849.6 | 4351.87887 |  | 7369 | 1441.07061 |  | 25243.4 | 4558.83268 |
| **4,4'-DDD** | 19841.4 | 2167.1324 |  | 10024 | 3531.41204 |  | 24606 | 3766.50091 |
| **p,p'-DDT** | 84955 | 18581.40703 |  | 10215.75 | 2766.34616 |  | 29975.6 | 9179.83216 |
| **Endrin ketone** | 63404.8 | 9975.65 |  | 21329 | 2806.86311 |  | 45655.2 | 9410.78231 |
| **Decachlorobiphenyl** | 144197.6 | 28024.25218 |  | 15540.5 | 10296.78389 |  | 64525.2 | 11740.78867 |
| **α-BHC** | 288479.4 | 35699.77981 |  | 93100.25 | 19642.88288 |  | 204610 | 32420.39254 |
| **Heptachlor** | 387846.8 | 50487.14922 |  | 117967.75 | 39051.93123 |  | 300112.2 | 52144.08952 |
| **Dieldrin** | 512969.8 | 67673.21094 |  | 226845 | 48297.63953 |  | 464411.8 | 70750.29168 |
| **α-Endosulfan** | 152.2 | 21.26358 |  | 154 | 47.75633 |  | 91.4 | 16.15735 |
| **Endrin aldehyde** | 28536.4 | 5430.46772 |  | 8322.75 | 1198.789 |  | 19685 | 2910.29698 |
| **Endosulfan sulfate** | 31209.6 | 4332.95761 |  | 8219.5 | 616.76123 |  | 15391.6 | 2640.6021 |
| **Methoxychlor** | 5425.4 | 1301.4365 |  | 260.75 | 143.82187 |  | 1266.4 | 465.19465 |
| **β-BHC** | 19448.6 | 2551.39122 |  | 6536.25 | 2141.78879 |  | 10693 | 2389.47314 |
| **α(cis)- Chlordane** | 78105.8 | 13113.25332 |  | 29555.75 | 8355.50839 |  | 65234 | 9558.97154 |

**Table S4. Temperature variation (5, 25, and 45 °C) for 21 organochlorine pesticides showing mean peak area and corresponding standard error.**

|  | **5°C** |  |  | **25°C** |  |  | **45°C** |  |
| --- | --- | --- | --- | --- | --- | --- | --- | --- |
| **Name of the pesticdes** | **Mean peak area** | **Standard error** |  | **Mean peak area** | **Standard error** |  | **Mean peak area** | **Standard error** |
| **γ- BHC** | 1334.66667 | 18.41497 |  | 78965 | 25445.95326 |  | 113443 | 35343 |
| **δ-BHC** | 1461 | 309.81338 |  | 256286.3333 | 46641.00848 |  | 298586 | 67417 |
| **Aldrin** | 772.66667 | 76.84255 |  | 150170.3333 | 26933.96744 |  | 198502 | 38942 |
| **Heptachlor epoxide** | 890 | 482.71834 |  | 101633.6667 | 17597.34077 |  | 134156.5 | 22876.5 |
| **γ (trans)- Chlordane** | 42 | 4.50925 |  | 69861 | 13062.37907 |  | 94254.5 | 17444.5 |
| **α (cis)- Chlordane** | 35.33333 | 21.75878 |  | 64896.66667 | 11503.64517 |  | 86727 | 14907 |
| **Endrin ketone** | 425.66667 | 248.89511 |  | 38096.33333 | 7837.29848 |  | 55341 | 10976 |
| **Decachlorobiphenyl** | 11.33333 | 5.48736 |  | 16791.66667 | 3934.65756 |  | 27285.5 | 8276.5 |
| **α-BHC** | 757.66667 | 380.57865 |  | 192647.3333 | 44167.76503 |  | 247504 | 61806 |
| **Heptachlor** | 1451 | 633.73838 |  | 332204.3333 | 65761.62735 |  | 447781.5 | 94636.5 |
| **Dieldrin** | 8739.66667 | 4535.1006 |  | 488082.3333 | 93572.73831 |  | 654222 | 115473 |
| **α.-Endosulfan** | 0 |  |  | 122.33333 | 24.88194 |  | 201.5 | 47.5 |
| **Endrin aldehyde** | 43.66667 | 9.40449 |  | 6643.33333 | 2192.25488 |  | 11603.5 | 3752.5 |
| **Endosulfan sulfate** | 64.33333 | 0.33333 |  | 14873.66667 | 2240.84466 |  | 20238.5 | 2776.5 |
| **Methoxychlor** | 13 | 9.50438 |  | 167.33333 | 45.57168 |  | 482 | 225 |
| **β.-BHC** | 232.33333 | 151.38949 |  | 6699.33333 | 2846.88135 |  | 8264 | 3243 |
| **p,p'-DDE** | 48.33333 | 0.33333 |  | 61539 | 15101.83684 |  | 90437 | 22336 |
| **Endrin** | 86 | 66.53069 |  | 18584.66667 | 3578.41037 |  | 26069 | 5100 |
| **β.-Endosulfan** | 60.33333 | 0.33333 |  | 21464.33333 | 4397.13006 |  | 29449 | 5810 |
| **4,4'-DDD** | 0 | 0 |  | 13883.33333 | 2838.08599 |  | 21077.5 | 3959.5 |
| **p,p'-DDT** | 0 | 0 |  | 20556 | 5124.41824 |  | 35428.5 | 10472.5 |

**Table S5. Comparative performance study between the developed TF-SPME patch and the commercial patch for 21 organochlorine pesticides showing mean peak area and corresponding standard error.**

|  | **Developed patch** |  |  | **Commercial patch** |  |
| --- | --- | --- | --- | --- | --- |
| **Name of the pesticdes** | **Mean peak area** | **Standard error** |  | **Mean peak area** | **Standard error** |
| **γ- BHC** | 62039.33333 | 21486.16699 |  | 101559.3333 | 15639.64774 |
| **δ.-BHC** | 216331 | 28748.81654 |  | 282052.3333 | 22111.67176 |
| **Aldrin** | 128817 | 22939.45315 |  | 179245.6667 | 16077.15077 |
| **Heptachlor epoxide** | 85172 | 13340.54738 |  | 113190 | 9432.78296 |
| **γ (trans)- Chlordane** | 58029.33333 | 10327.42111 |  | 75707.66667 | 6419.27032 |
| **α (cis)- Chlordane** | 53419.66667 | 9124.28591 |  | 70302.66667 | 5590.12323 |
| **p,p'-DDE** | 50133.33333 | 15217.8851 |  | 67975.66667 | 7935.8577 |
| **Endrin** | 15951.66667 | 3028.70337 |  | 20178.33333 | 2134.37652 |
| **β-Endosulfan** | 17309 | 3577.20566 |  | 22052.66667 | 1994.49746 |
| **4,4'-DDD** | 5221 | 3207.35421 |  | 5253.33333 | 1181.0366 |
| **p,p'-DDT** | 25491 | 5948.11107 |  | 21202 | 1412.31488 |
| **Endrin ketone** | 32899.66667 | 8450.38982 |  | 40336 | 2745.09696 |
| **Decachlorobiphenyl** | 16113 | 5820.56263 |  | 21461.33333 | 1656.50559 |
| **α-BHC** | 162719 | 28778.66738 |  | 221929 | 21353.86207 |
| **Heptachlor** | 280987 | 50327.04354 |  | 370259.6667 | 39344.3884 |
| **Dieldrin** | 404008 | 72465.76228 |  | 544527.6667 | 49268.96489 |
| **α-Endosulfan** | 129.33333 | 24.77454 |  | 139 | 6.65833 |
| **Endrin aldehyde** | 13550.66667 | 2999.06321 |  | 14723 | 1513.36083 |
| **Endosulfan sulfate** | 13481.66667 | 3179.33989 |  | 12495.66667 | 648.30274 |
| **Methoxychlor** | 225.66667 | 125.77272 |  | 60.33333 | 37.37349 |
| **β-BHC** | 5743.66667 | 2501.5053 |  | 7727 | 1551.32277 |

**Effect of pH**

To investigate the effect of pH on the extraction efficiency using the DVB-coated TF-SPME patches, the OCPs were extracted from the sample matrix consisting of three pH individual conditions: an acidic (pH 3), neutral (pH 7), and basic (pH 11) media. Neutral pH (pH 7) yielded the highest extraction efficiency for most OCPs, followed by acidic pH (pH 3), whereas a lower extraction response was observed at basic pH (pH 11). This phenomenon could be explained by the hydrophobic and non-ionizable characteristics of organochlorine pesticides. The OCPs mostly remained in the molecular form (non-dissociated) at acidic and neutral pH, and this can enhance the interaction of analytes with the hydrophobic DVB sorbent particles through π–π and hydrophobic interactions. In addition, the hydrogen bonding also plays a minor role for the moderately polar analytes. At high pH, extraction efficiency was significantly reduced due to increased hydroxide ion concentration in the sample matrix as it could disrupt the interaction between the sorbent and the analytes. Therefore, the optimal extraction condition was chosen as pH 7 for getting the maximum adsorption (Fig S2).


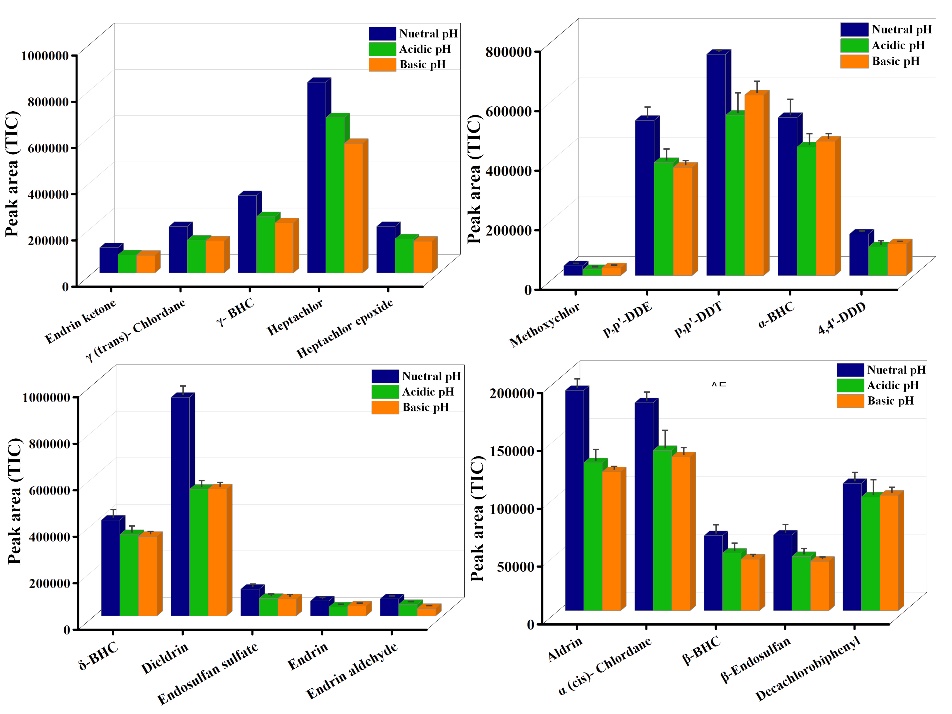


Fig S2: Effect of pH: Assessment of pH conditions on extraction efficiency

**Effect of Salt**

To investigate the influence of ionic strength, the extraction efficiency of the target OCPs was studied by adding NaCl in the concentration range from 0% to 12% (w/v). As presented in Fig. S3, the extraction efficiency of pesticides increased with salt addition until 6% NaCl and then decreased gradually. Such behaviour is generally regarded as the salting-out effect. The presence of added salt decreases the solubility of non-polar analytes in the aqueous phase. Both Na⁺ and Cl⁻ ions compete with the analyte molecules for solvation by water molecules and, therefore, decreases the amount of water molecules available for the solvation of the pesticides. Thus, analytes partition into the hydrophobic DVB/PDMS coating, resulting in increased extraction efficiency and high peak areas at up to 6% NaCl. At higher concentrations above 6%, extraction efficiency declined because of the increased viscosity of the sample matrix. This reduces the diffusion of analyte molecules, thereby limiting the transport of the analyte from the bulk phase to the extraction surface. Excessive ionic strength can alter the equilibrium distribution between the aqueous and sorbent phases, thereby decreasing analyte transfer. Therefore, a moderate concentration of NaCl at 6% exhibits maximum extraction efficiency by maintaining a balance between salting out and diffusivity in the solution
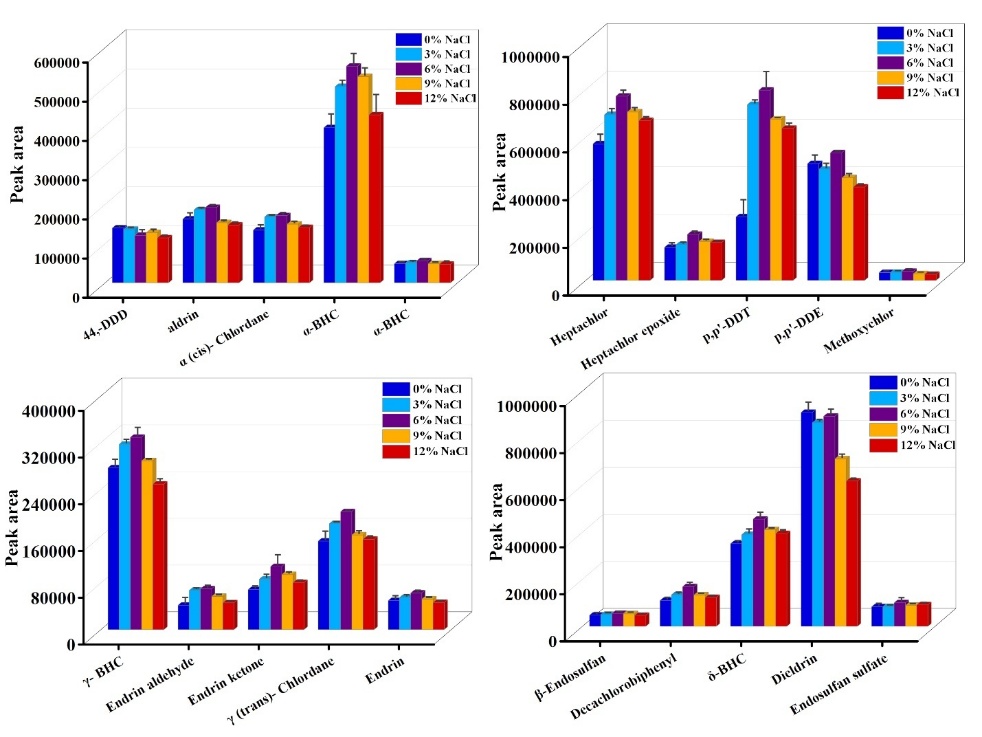


Fig S3: Effect of salt: Assessment of NaCl variation on extraction efficiency

**Table S6**. Comparison of various extraction techniques for the determination of organochlorine pesticides (OCPs) in water.

This table summarizes extraction based approaches reported for the analysis of organochlorine pesticides (OCPs) in aqueous matrices. Various sorbent materials, including composite fibers, metal organic frameworks, ionic liquids, graphene-based coatings, and capsule-phase systems, are highlighted along with the extraction conditions, desorption modes, sensitivity, and reusability

| Study / Year | Extraction Technique / Coating | Target analytes (OCPs) | Extraction Time (min) | Desorption Solvent & Volume | LOD (ng/mL or ng/L) | Reusability | Reference |
| --- | --- | --- | --- | --- | --- | --- | --- |
| C18 composite SPME fiber / 2021 | DI-SPME (fiber) / C18–silicone composite coating on stainless steel wire | HCB, trans-chlordane, cis-chlordane, o,p-DDT, p,p-DDT, mirex | 60 min | Thermal desorption (solvent-free) | 0.059–0.151 ng/L | Reusable, good thermal & solvent stability | [1] |
| Quinoline-linked 2D COF / 2024 | HS-SPME (fiber) / Quinoline-linked ultrastable 2D COF | HCH isomers, DDTs, endrin, aldrin, heptachlor | 30 min | Thermal desorption (solvent-free) | 0.0005–0.013 ng/mL | Highly reusable (>120 cycles) | [2] |
| Ionic-liquid SB-µ-SPE / 2023 | Stir-bar membrane-protected µ-SPE / ionic liquid sorbent ([4-MA][FeCl₄], [1-NA][FeCl₄]) | 19 OCPs including lindane isomers, aldrin, endrin, methoxychlor | 15 min | Hexane:toluene mixture, 200 µL (ultrasound 30 min) | 0.25–3.4 ng/mL | Not reported | [3] |
| Sol–gel graphene fiber / 2012 | SPME fiber / Sol–gel graphene coating on steel wire | HCB, heptachlor, aldrin, trans-chlordane, endrin, o,p-DDT, p,p-DDT | 25 min | Thermal desorption (250 °C, 5 min) | 0.19–18.3 ng/L | Reusable, stable to 350 °C | [4] |
| AuNP–SPME / 2020 | DI-SPME fiber / AuNPs layer-by-layer (gallic acid / H₂O₂) | Heptachlor, dieldrin, methoxychlor, DDTs, endosulfan isomers | 60 min | Thermal desorption (260 °C, 3 min) | 130 ng/L | >100 extractions | [5] |
| PEG-300 CPME / 2024 | Capsule Phase Microextraction / PEG-300 sol-gel capsule | HCHs, Aldrin, DDEs, DDDs, methoxychlor | 50 min | 250 µL acetone | 0.01–0.03 ng/mL | Reusable; no carryover | [6] |
| PSE → SPE → SPME (PDMS fiber) / 2016 | SPME – PDMS fiber (100 µm) used after SPE cleanup | OCPs within mixture of 103 pesticides | 40 min (at 80 °C) | Ethyl acetate + toluene + ACN; final in 1 mL ACN | 0.01–153.2 ng sampler⁻¹ | Not reusable (passive sampler processing) | [7] |
| Bursting-bubble flow ME / 2022 | BBFME (solvent-less microextraction, no coating) | Simazine, γ-BHC, Alachlor, Aldrin, Dieldrin | 6 min | Solvent-free (direct aerosol collection) | 0.24–0.33 µg/L (240–330 ng/L) | Not reusable | [8] |
| **Assessment of Greenness score of the technique using CACI, CaFRI, AGSA Tools**  To assess the environmental sustainability, the proposed method was analyzed with several green analytical tools to quantify the eco-friendliness**.** Along with AGREE, ComplexMoGAPI and BAGI, the Click Analytical Chemistry Index (CACI), the Carbon Footprint Reduction Index (CaFRI) and the Analytical Green Star Area (AGSA) scores were calculated.  The Click Analytical Chemistry Index (CACI)[9] output provides a graphical representation of the method's practicality and overall performance, yielding a score of 77. The CACI provided the information about sample size, sample preparation, feasibility, scope of application, portability, automation, and sensitivity. This score reflects a well-balanced method with excellent applicability, reasonable feasibility, and efficient operational characteristics so that the method is suitable for routine analytical uses (Fig S4a & Table S7). The Carbon Footprint Reduction Index (CaFRI) [10]is a specialized sustainability assessment tool designed to quantify and compare the carbon footprint associated with analytical methods. It evaluates the energy consumption, waste generation, transportation requirements, reagent usage, and overall operational practices involved in an analysis to determine how effectively a method minimizes greenhouse gas emissions (Fig S4b & Table S8). The Analytical Green Star Area (AGSA)[11] is a modern evaluation tool created to assess the environmental sustainability of analytical methods. Based on the 12 Principles of Green Analytical Chemistry, AGSA quantifies a method's compliance with each principle. AGSA provides a visual "green star" diagram in which each point represents a principle, enabling the user to identify strengths and areas that need further improvement. The parameters involve sample size, waste produced, energy consumption, and might help to further optimize the methods toward greener, more efficient, and sustainable analytical practices (Fig S4c & Table S9).  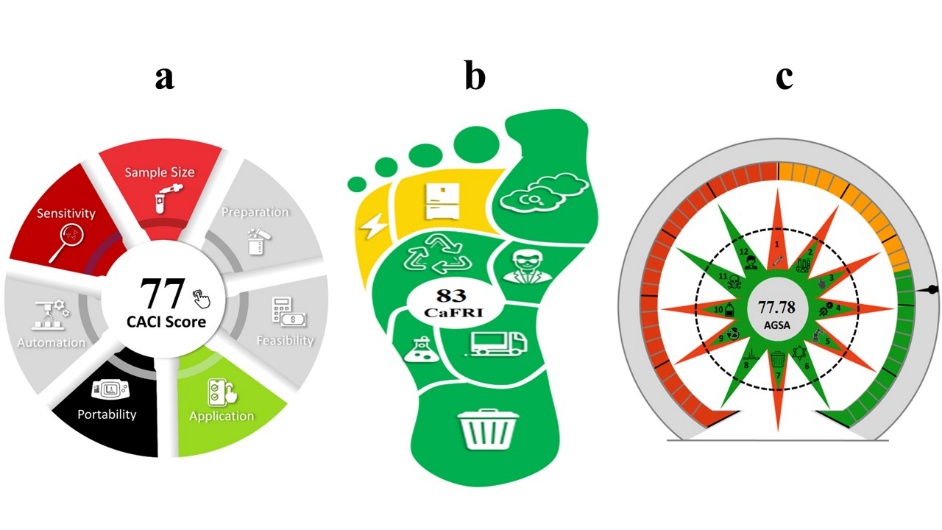  Fig S4. Green Analytical Assessment of the Developed TF-SPME Method Using (a) CACI, (b) CaFRI, (c) AGSA Tools  Table S7 : Depicts the parameters and corresponding values used for calculating the CACI score for the developed analytical method.   \| **Parameter** \| **Response** \| \| --- \| --- \| \| Sample Size \| < 1 mL / g \| \| Sample Preparation Level \| Minimal sample preparation \| \| Sample Preparation Time (min) \| 5 \| \| Chemicals & Reagents Availability \| Not commercially available but easily synthesized \| \| Instrument Availability \| All equipment available in regular analytical labs \| \| Cost per Sample \| < $10 per sample \| \| Application Type \| Quantitative \| \| Number of Analytes Tested \| > 3 analytes \| \| Number of Matrices Tested \| > 3 matrices \| \| Portability of Instrumentation \| Not portable nor miniaturized \| \| Automation Level \| Semi-automatic \| \| Sensitivity Level \| < 1% target concentration \|  \| **Parameter** \| **Response** \| \| --- \| --- \| \| 1. Energy reduction program or clean energy sources adapted \| No \| \| 2. Total electrical power use of analytical instruments \| 0.1–1.5 kW \| \| 3. Energy-intensive non-analytical equipment essential \| No \| \| 4. Number of samples analyzed per hour \| <10 samples/h \| \| 5. Carbon footprint of electrical power known \| Yes \| \| 6. Emission factor \| 0.1–0.3 kg CO₂/kWh \| \| 7. Sample storage \| Storage under normal conditions \| \| 8. Sample transportation required \| No \| \| 9. Distance between sample field and laboratory \| Not applicable \| \| 10. Number of samples transported per shipment \| Not applicable \| \| 11. Eco-friendly vehicle used \| Not applicable \| \| 12. Personnel required per sample analysis \| 1 person \| \| 13. Automation level \| Semiautomatic \| \| 14. Waste amount \| <10 mL or g per sample \| \| 15. Waste disposal \| Specialized personnel \| \| 16. Recycling \| Recycled reagents/solvents used from same method \| \| 17. Total number of pictograms \| ≤3 \| \| 18. Total organic solvents per sample \| <5 mL \| \| 19. Total reagents per sample \| <1 g or mL \|   Table S8: Represents the parameters and corresponding values used for calculating the score for CaFRI the developed technique.  Table S9: Represents the parameters and corresponding values used for calculating the AGSA score for the developed analytical method.   \| Principle \| Question \| Answer \| \| --- \| --- \| --- \| \| Principle 1 (Direct Analysis) \| Extent of sample treatment required \| Extensive treatment (e.g., extraction, filtration, concentration) \| \| Principle 2 (Minimum Sample Size) \| Sample size required \| Between 0.1 gram and 1 gram \| \| Principle 3 (In-Situ Measurements) \| Where measurements are performed \| Samples are analyzed in a mobile lab or field lab \| \| Principle 4 (Integration of Processes) \| Level of integration \| Some processes integrated, reducing steps and instrument use \| \| Principle 5 (Automation & Miniaturization) \| Degree of automation \| Semi-automated \| \|  \| Degree of miniaturization \| Fully miniaturized \| \| Principle 6 (Avoid Derivatization) \| Derivatization required? \| No derivatization required \| \| Principle 7 (Waste & Management) \| Volume of waste generated \| Between 100 mL and 1 L per sample \| \|  \| Waste management approach \| Waste recycling with reuse and sustainable practices \| \| Principle 8 (High Throughput) \| Analytes per run \| More than 3 analytes \| \| Principle 9 (Low Energy Consumption) \| Energy consumption \| 1–1.5 KW/sample \| \| Principle 10 (Renewable Reagents) \| Source of reagents \| Mixture of renewable and non‑renewable reagents \| \| Principle 11 (Low Toxicity) \| Toxicity level \| 2 pictograms or less \| \| Principle 12 (Operator Safety) \| Safety level \| Low-risk procedures with minimal PPE needed \|   Characteristic functional groups of both PDMS and DVB were confirmed by the FT-IR spectrum of the DVB/PDMS-coated TF-SPME patch. The weak band at 2900 cm⁻¹ corresponded to the aliphatic C-H stretching of Si-CH₃ groups in PDMS. The strong absorption at 1260 cm⁻¹ has been attributed to Si-CH₃ bending, while the intense peak at 1015 cm⁻¹ arose from the asymmetric stretching of the Si-O-Si backbone, the most salient feature of PDMS. The peak present at 794 cm⁻¹ was linked to Si-C stretching and CH₃ rocking vibrations. These characteristic vibrational bands confirmed that the carbon-fiber substrate was successfully coated with the DVB/PDMS polymeric layer (Fig S5)  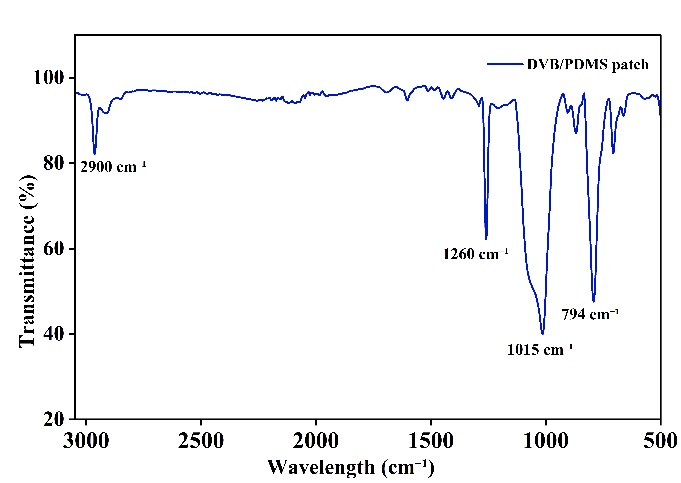 | | | | | | | |

Fig S5: Fourier Transform Infrared Spectra (FT-IR) of synthesized DVB/PDMS patch.

Table S10. Base Peak and Fragment Ions (m/z) used for GC–MS identification of the 21 OCP

| **Compound** | **Base peak** | **Fragmented peaks** |
| --- | --- | --- |
| δ-BHC | 146.9 | 182.8 |
| γ-BHC | 146.9 | 82.9, 182.8, 108.9 |
| α-BHC | 182.9 | 144.8, 180.8, 218.8 |
| Decachlorobiphenyl | 178.9 | 427.7, 497.5, 213.8 |
| Methoxychlor | 213 | 185, 197.1 |
| Endrin ketone | 41.1 | 100.9, 280.8 |
| \|  \| 4,4′-DDD \| \| --- \| --- \| | 165.1 | 200.1, 165.1, 237 |
| p,p′-DDE | 176 | 211, 245.8 |
| Endosulfan sulfate | 236.8 | 386.8, 288.8 |
| \|  \| Endrin aldehyde \| \| --- \| --- \| | 41.1 | 65, 67 |
| α-Endosulfan | 159 | 194.9, 125 |
| p,p′-DDT | 165.1 | 200, 164.9, 234.8 |
| \|  \| β-Endosulfan \| \| --- \| --- \| | 158.9 | 194.9 |
| \|  \| Aldrin \| \| --- \| --- \| | 65 | 40.1, 66 |
| \|  \| Heptachlor \| \| --- \| --- \| | 65 | 236.8, 99.9, 271.8 |
| \|  \| Dieldrin \| \| --- \| --- \| | 77 | 78.9 |
| \|  \| α-(cis) Chlordane \| \| --- \| --- \| | 265.8 | 336.8, 300.8, 372.7 |
| γ-(trans) Chlordane | 265.8 | 300.8, 372.7 |
| Heptachlor epoxide | 262.8 | 53.1, 316.8, 352.7 |
| \|  \| Endrin \| \| --- \| --- \| | 53.1 | 80.9 |
| β-BHC | 75 | 84.9, 110.9 |

**Reference**

1. Li, S., Lu, C., Zhu, F., Jiang, R. & Ouyang, G. Preparation of C18 composite solid-phase microextraction fiber and its application to the determination of organochlorine pesticides in water samples. *Anal Chim Acta* 873, 57–62 (2015).

2. Yang, Y. *et al.* An ultrastable 2D covalent organic framework coating for headspace solid-phase microextraction of organochlorine pesticides in environmental water. *J Hazard Mater* 452, 131228 (2023).

3. Abdi Hassan, A., Sajid, M., Al Ghafly, H. & Alhooshani, K. Ionic liquid-based membrane-protected micro-solid-phase extraction of organochlorine pesticides in environmental water samples. *Microchemical Journal* 158, 105295 (2020).

4. Ke, Y. *et al.* Preparation of graphene-coated solid-phase microextraction fiber and its application on organochlorine pesticides determination. *J Chromatogr A* 1300, 187–192 (2013).

5. Gutiérrez‐Serpa, A., Rocío‐Bautista, P., Pino, V., Jiménez‐Moreno, F. & Jiménez‐Abizanda, A. I. Gold nanoparticles based solid‐phase microextraction coatings for determining organochlorine pesticides in aqueous environmental samples. *J Sep Sci* 40, 2009–2021 (2017).

6. Ferracane, A. *et al.* Monolithic capsule phase microextraction prior to gas chromatography-mass spectrometry for the determination of organochlorine pesticides in environmental water samples. *Microchemical Journal* 186, 108355 (2023).

7. Levy, M., Al-Alam, J., Delhomme, O. & Millet, M. An integrated extraction method coupling pressurized solvent extraction, solid phase extraction and solid-phase micro extraction for the quantification of selected organic pollutants in air by gas and liquid chromatography coupled to tandem mass spectrometry. *Microchemical Journal* 157, 104889 (2020).

8. Tazarv, M., Faraji, H., Moghimi, A. & Azizinejad, F. Bursting bubble flow microextraction combined with gas chromatography for determination of organochlorine pesticides in aqueous samples. *Microchemical Journal* 181, 107793 (2022).

9. Mansour, F. R., Bedair, A. & Locatelli, M. Click Analytical Chemistry Index as a novel concept and framework, supported with open source software to assess analytical methods. *Advances in Sample Preparation* 14, 100164 (2025).

10. Mansour, F. R. & Nowak, P. M. Introducing the carbon footprint reduction index (CaFRI) as a software-supported tool for greener laboratories in chemical analysis. *BMC Chem* 19, 121 (2025).

11. Mansour, F. R., Bedair, A., Belal, F., Magdy, G. & Locatelli, M. Analytical Green Star Area (AGSA) as a new tool to assess greenness of analytical methods. *Sustain Chem Pharm* 46, 102051 (2025).
